# Experimental and advanced equilibrium studies on the enhanced adsorption of phosphate, cadmium, and safranin dye pollutants using methoxy exfoliated glauconite

**DOI:** 10.3389/fchem.2024.1471994

**Published:** 2024-11-06

**Authors:** Mostafa R. Abukhadra, Aya Fadl Allah, Mohamed Shaban, Noof A. Alenazi, Haifa A. Alqhtani, May Bin-Jumah, Ahmed A. Allam, Stefano Bellucci

**Affiliations:** ^1^ Materials Technologies and their Applications Lab, Geology Department, Faculty of Science, Beni-Suef University, Beni Suef, Egypt; ^2^ Geology Department, Faculty of Science, Beni-Suef University, Beni Suef, Egypt; ^3^ Department of Chemistry, Faculty of Science, Beni-Suef University, Beni-Suef, Egypt; ^4^ Department of Physics, Faculty of Science, Islamic University of Madinah, Madinah, Saudi Arabia; ^5^ Department of Chemistry, College of Science and Humanities in Al-Kharj, Prince Sattam bin Abdulaziz University, Al-kharj, Saudi Arabia; ^6^ Department of Biology, college of Science, Princess Nourah bint Abdulrahman University, Riyadh, Saudi Arabia; ^7^ Department of Biology, College of Science, Imam Mohammad Ibn Saud Islamic University, Riyadh, Saudi Arabia; ^8^ Department of Zoology, Faculty of Science, Beni-suef University, Beni-suef, Egypt.; ^9^ INFN-Laboratori Nazionali di Frascati, Frascati, Italy

**Keywords:** glauconite, exfoliation, methoxy, pollutants, adsorption, advanced equilibrium

## Abstract

Natural glauconite, as a mixed-layered clay mineral, was subjected to exfoliation processes, producing silicate monolayers or individual sheets that were further modified with methanol into methoxy exfoliated glauconite (Mth/EXG). The structure was assessed as an enhanced adsorbent for three types of common water contaminants, including phosphate (PO_4_^3-^), safranin-O dye (SFR), and cadmium metal ions (Cd^2+^). The Mth/EXG structure achieved promising adsorption capacities at the saturation points equal to 269.9 mg/g for PO_4_^3-^, 312 mg/g for SFR, and 234.5 mg/g for Cd^2+^ which are significantly better than the reported values for several studied adsorbents of higher costs and complex production procedures. The adsorption processes and the predicted regulated mechanisms in terms of the adsorbate/adsorbent interface were illustrated based on the steric and energetic findings that correspond to the applied monolayer equilibrium model of one energy site. The structure displays active site densities of 82.5 mg/g (PO_4_^3-^), 136.3 mg/g (SFR), and 83.4 mg/g (Cd^2+^), which illustrate the high uptake performance of SFR. Also, the steric parameters reflected the suitability of each existing site to be filled with 4 ions of PO_4_^3-^, SFR, and Cd^2+^. The adsorption energy (less than 40 kJ/mol) in conjunction with free adsorption energy from D-R model (8–16 kJ/mol) and steric parameters validate the dominant impact of the multi-ionic physical mechanisms (hydrogen bonding and van der Waals forces), in addition to the assistant impact of some weak chemical processes that might be assigned to the formed inner-sphere complex. Also, these reactions all occurred spontaneously with exothermic behaviors according to the thermodynamic functions. Additionally, the structure exhibit significant affinity for the studied pollutants even in the existing of completive chemical including anions, cations and organic molecules.

## 1 Introduction

The greatest risk confronting modern civilization is the contamination of drinkable water and the protection of its population (Hsu et al., 2024; Zourou et al., 2022). The World Health Organization (WHO) has released a grave alert, forecasting that by 2025, over 50% of the worldwide population will suffer from significant water shortages (Zourou et al., 2022; Arab et al., 2022). Industrial as well as the other activities of agriculture and mining emit numerous water contaminants, such as bacteria, herbicides, toxic metals, chemical fertilizers, medicinal residues, and dyes (Ighnih et al., 2024; Kusuma et al., 2024). The discharge of phosphate ions (PO_4_^3-^) through water sources, particularly enclosed lakes, at levels as high as 0.05 mg/L has a notable negative impact on the water’s purity along with the aquatic ecosystems (Wu et al., 2024; Saleh et al., 2024; Shi et al., 2019). The unregulated release of phosphate promotes the unrestricted proliferation of phytoplankton and algae, ultimately causing the occurrence of eutrophication (Shi et al., 2019; Che et al., 2024). Moreover, the existence of heavy metals in water supplies as water-soluble ions has been identified as harmful and malignant contaminants that have a significant tendency to become accumulated inside human beings, cells, and tissues (Salam et al., 2020; Jiang et al., 2020). Cadmium ions (Cd^2+^) are very hazardous and should not exceed a concentration of 0.003 mg/L in drinking water (Vilela et al., 2019). In addition, Cd^2+^ contaminants have many adverse health impacts, including pulmonary edema, acute disorder, chronic disorder, itai-itai illness, emphysema, hepatic failure, high blood pressure, testicular atrophy, kidney failure, and osteomalacia (Souza-Arroyo et al., 2022; Rahman and Raheem, 2024). Also, the Cd^2+^ ions have a significant inhibitory influence on the growth of seeds, crop length, root extension, and leaf production (Rasafi et al., 2020). Furthermore, the presence of high levels of Cd^2+^ has detrimental impacts on aquatic organisms and the surrounding environment, as well as the commercial value of fish (Abdellaoui et al., 2024).

The synthetic dyes comprise a broad group of aromatic chemicals that are frequently utilized as essential pigments in many sectors, such as textiles, plastic, paper, and leather (Ighnih et al., 2023a; Neolaka et al., 2024). Every year, around 700,000 tons of produced dyes are expected to be discharged throughout the nearby areas alongside aquatic habitats (Pathania et al., 2021). Most commercially developed dyes have been identified as poisonous and highly resistant against biodegradability, resulting in detrimental effects on habitats and human wellbeing (Arab et al., 2022; Pathania et al., 2021). Safranin dye (SFR) is a form of basic azine dye that dissolves in water easily and is frequently employed for printing fabrics, staining, microbial identification, medicine, and food containers (Ghosh et al., 2021; Shaltout et al., 2022). The intricate composition and stability of SFR pose challenges throughout its biological decomposition (Shaltout et al., 2022). This dye possesses the capacity to eradicate the nucleic acid within bacteria while also demonstrating tumor-promoting and carcinogenic characteristics. Extended or brief exposure to SFR could cause many adverse health consequences, which involve inflammation of the eyes, lips, tongue, and stomach in addition to redness and itching of the skin. Other symptoms related to being exposed to SFR include feelings of nausea, vomiting, and discomfort in the digestive system (Ghosh et al., 2021; De Araujo et al., 2023).

Researchers have acknowledged advanced oxidation, ozonation, adsorption, flocculation, nanofiltration, membrane separation, biological degradation, ionic exchange, and coagulation as efficient methods for remediating different species of pollutants (Hsu et al., 2024; Saleh et al., 2024; Jiang et al., 2020). However, clogged membranes often cause problems for various filtering systems by sealing off their internal pores. This consequently increases pressure and power consumption, shortens the membranes’ lifespan, and renders the method extremely costly. However, it is recommended to use adsorption approaches to reduce the harmful effects of the resulting chemical intermediates during the oxidation and catalytic decomposition processes (Neolaka et al., 2023a; Wu et al., 2023). Numerous studies indicate that adsorption using new materials is an affordable, effective, safe, readily available, and recoverable method for the decontamination of diverse water contaminants (Naat et al., 2021; Wang et al., 2024). The selection of a successful adsorbing material is affected by various factors, involving production expenses, production processes, precursor availability, adsorption efficiency, recyclability, uptake capacity, biodegradability, uptake specificity, safety, durability, and reactivity (Fan et al., 2024; Khattari et al., 2024). As a result, a thorough evaluation has been performed to develop novel adsorbents using accessible and economical materials frequently encountered in the earth’s resources (Hsu et al., 2024; Ifa et al., 2022; Hamidi et al., 2024). The use of recognized adsorbents produced from earth’s resources, such as various minerals and rocks, is strongly advocated because of their significant environmental and economic benefits (Chen et al., 2021).

The application of functional clay-based nanomaterials has been established to be efficient in safely removing various organic and inorganic contaminants, providing the advantages of affordability as well as environmental sustainability (Alqahtani et al., 2023; Yao et al., 2024; Ahmed et al., 2024a). Clay minerals are frequently composed of flexible frameworks constructed of numerous layers of aluminosilicates. These minerals possess a tendency to exchange ions, exhibit strong chemical stability, display a reactive interface that is beneficial for chemical reactions, possess an excellent potential for adsorption, and are resistant to high temperature levels (Alqahtani et al., 2023; Ighnih et al., 2023b; Cigeroglu et al., 2024). Additional studies were accomplished to improve the surface chemistry and physicochemical characteristics of frequently utilized clay minerals by several modification techniques. The main objective underlying such studies is to explore their potential as adsorbents to remove inorganic and organic ions (Ighnih et al., 2023b; Meng et al., 2020). These techniques include alkaline chemical treatments, thermal processing, acidic activation, pillaring with metal ions, blending with metal oxides, exfoliating, integrating with polymers, scrolling, and organic hybridization by employing chemicals involving CTAB along with starch (Ighnih et al., 2023b; Meng et al., 2020; Ighnih et al., 2023c).

Methoxy-modified structures have recently been reported to be innovative varieties of modified clay, displaying improved adsorption qualities (Ashiq et al., 2021; Tan et al., 2018). By employing eco-friendly grafting approaches, the alcoholic molecules can intercalate with the structurally layered units of clays, which are saturated with numerous hydroxyl groups, particularly across the interior interface (Tan et al., 2018; Tchoumene et al., 2022; Li et al., 2017). The grafting of methanol within the silicate layers of clay resulted in significant enhancement in the surface area, dispersion behavior, the surface reactivity, the surficial charges, and the quantities of the existed active sites or centers (Ashiq et al., 2021; Li et al., 2017). Therefore, the methoxy modified versions of clay were applied effectively in the removal of several species of water contaminants including oxytetracycline, bisphenol, dyes, and heavy metals (Ashiq et al., 2021). The prior studies have mostly examined the technical characteristics of methoxy forms of kaolinite together with bentonite. However, there has been a lack of investigation into the distinctive characteristics of the methoxy derivatives that include different clay minerals, such as glauconite (Ashiq et al., 2021; Borah et al., 2023).

Glauconite is a prevalent clay mineral encountered in nature, characterized by its chemical framework as a potassium-ferric phyllosilicate (K, Na) (Fe^3+^Fe^2+^, Al, Mg)_2_(Si,Al)_4_O_10_(OH)_2_). Glauconite is comprised of alternating layers comprised of illite and smectite, with an alumina di-octahedral subunit sandwiched inside two silica tetrahedron subunits. These successive layers also include cations of potassium inside the interlayer spaces. As a mineral, glauconite possesses extensive natural availability, affordable expenses, a metal-enriched chemical framework, an appealing geometry, an excellent surface area, potential catalytic characteristics, and strong ion exchange efficiency (Singla et al., 2020; Shmandiy et al., 2020). Consequently, it has a great number of interchangeable hydroxyl groups that facilitate the incorporation of alcoholic molecules into its multilayered framework. This attribute enables the methanol to bond without requiring further pre-treatment procedures (Wang et al., 2024; Meng et al., 2020). As a consequence, it was expected that integrating methanol within the glauconitic layers could yield a multifunctional hybridized structure exhibiting improved physicochemical characteristics (Borah et al., 2023). Moreover, in the last few years, significant progress has been made in the investigation of the morphological modifications of clay, specifically in the area of splitting or scrubbing the structurally layered units as independent silicate layers with two-dimensional geometrical features (Alqahtani et al., 2023; Abdo et al., 2022). The application of this method has resulted in unique and innovative clay-based nanostructures. These frameworks exhibit distinctive characteristics such as powerful biological activities, adsorption capacity, oxidization qualities, exterior reactive properties, dispersion behaviors, and surface area (Das et al., 2023; Tang et al., 2023; Abukhadra et al., 2019; Baldermann et al., 2022).

It was expected that the synthesis of methoxy types of the separated and exfoliated glauconite nano-sheets is expected to result in very effective adsorbents with enhanced physicochemical properties and adsorption capacities for a variety of water contaminants. As a result, the presented investigation included the first investigation of methoxy exfoliated glauconite (Mth/EXG) as enhanced adsorbent for different species of common water pollutants including phosphate ions (PO_4_^3-^), safranin dye (SFR), and cadmium metal ions (Cd^2+^). The adsorption behaviors and effective mechanistic processes were evaluated for the first times employing standard experimental factors, kinetic modeling, and classic isotherm approaches, and advanced equilibrium modeling according to aspects of statistical physics theory. The parameters that are under analysis encompass the saturation levels, retention capacities, actual density of occupied sites, quantity of pollutant ions bound on a single site, binding energies, and thermodynamic characteristics.

## 2 Experimental work

### 2.1 Materials

The glauconite mineral was purchased from the El-Gedida location at the El-Bahariya Oasis, in the Western Desert of Egypt. The analyzed sample exhibited a chemical structure consisting of SiO_2_ (52.2%), Al_2_O_3_ (6.12%), Fe_2_O_3_ (23.14%), SO_3_ (0.17%), K_2_O (6.48%), Na_2_O (0.08%), MgO (3.53%), TiO_2_ (0.11%), CaO (0.27%), P_2_O_5_ (0.10%), MnO (0.01%), and 7.8% loss on ignition (L.O.I.). The chemical analysis was conducted using Panalytical Axios Advanced XRF technique, Nuclear Material Authority, Egypt. The chemicals used during the glauconite conversion procedure were obtained from Sigma-Aldrich in Egypt. These included cetyltrimethylammonium bromide (CTAB) with a purity of over 98%, dimethyl sulfoxide (DMSO) with a purity of over 99.5%, sodium hydroxide (NaOH) with a purity of 97%, and methanol with a purity of over 99.9%. The adsorption experiments involved safranin-O synthetic dye (≥85%; Sigma-Aldrich), together with phosphate and cadmium standard solutions (1,000 mg/L; Sigma-Aldrich) as the primary sources of the polluted solutions.

### 2.2 Synthesis of methoxy exfoliated glauconite (Mth/EXG)

**FIGURE 1:**
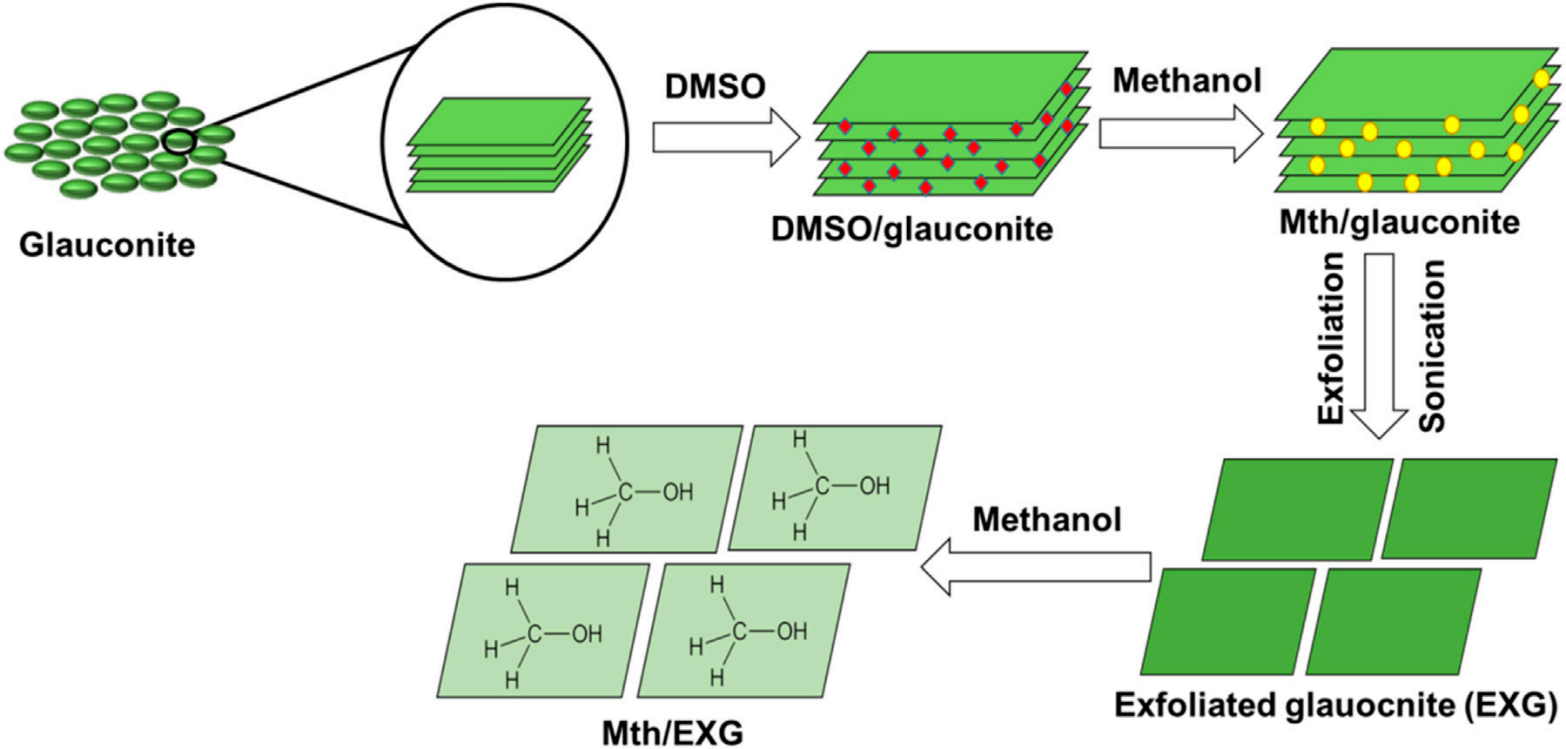
Schematic diagram for the synthesis procedures of Mth/EXG structure.

The EXG was produced using the published procedures established by Abukhadra et al. (2024). About 40 g of the original glauconite, as a crude mined material, was extensively ground and then combined with 200 mL of a dilute DMSO mixture (80% DMSO and 20% water). The combination was agitated rapidly for 72 h. This approach is crucial during this preparation stage to destruct the existing chemical bonds, specifically the hydrogen bonds connecting subsequent layers of clay, specifically the illite subunits present in the heterogeneous structure of glauconite with smectite. The resultant product was subjected to five methanol rinsing rounds, each lasting about 20 min. This procedure led to the development of methoxy glauconite, exhibiting significant exfoliating of its crystalline layers (Mth/EXG) and organophilic properties. Subsequently, the Mth/EXG particles were removed by filtration employing Whitman filter paper, repeatedly rinsed with distilled water, and dried at 60 °C. Then the sample was immersed in methanol for an additional 48 h to ensure the formation of methoxy-modified forms (Mth/EXG) (Figure 1).

### 2.3 Characterization instruments

The material’s structure and crystalline characteristics were evaluated and examined by analyzing their X-ray diffraction (XRD) characteristics employing a PANalytical-Empyrean X-ray diffractometer with Cu Kα radiation, operated at 20 mA and 40 kV in the 2θ range of 0–70 at a scanning speed of 5°/min. The Shimadzu FTIR-8400S spectrometer has been employed to evaluate the alteration in the basic chemical groups throughout the fabrication process. The spectrometer has a detection spectrum of 400–4,000 cm^-1^. The surface features of the formed derivatives, alongside the original glauconite, have been examined using a Gemini Zeiss Ultra 55 scanning electron microscope. Prior to imaging, the exteriors of the ingredients under study had been prepared by coating them with thin gold films. Furthermore, the inner structures were then examined utilizing HRTEM images, which were obtained by a transmission electron microscope (JEOL-JEM2100) at an acceleration voltage of 200 kV. The porosity levels and specific surface area were measured using a surface area analyzer (Beckman Coulter SA3100) just after the samples were degassed. The evaluation has been completed using the standard N_2_ adsorption and desorption isotherms.

### 2.4 Adsorption studies

PO_4_^3-^, SFR, and Cd^2+^ adsorption experiments have been performed in batches. The assessed experimental factors involved the effects of pH (pH 2–8), interaction duration (20–780 min), Mth/EXG quantity (0.2 g/L), and pollutants concentrations (50–400 mg/L) at three distinct operating temperature settings (20°C, 30°C, and 40 °C) and specific volumes (100 mL). Every test included three iterations, and the means of the results attained have been implemented throughout the computations, having standard deviations less than 4.12% (PO_4_^3-^), 4.36% (Cd^2+^), and 3.75% (SFR). Following the adsorption investigations, the resulting solutions after uptake of Cd^2+^ had been acidified using 2% HNO_3_, and the remaining Cd^2+^ ions have been measured using inductively coupled plasma optical emission spectrometry (ICP-OES) (Avio 220 Max ICP-OES; Perkin Elmer). The binding capacities (Qe) of the investigated ions were determined using Equation 1. The determination of the rest PO_4_^3-^ had been conducted employing the Dionex DX-120 ion chromatography device. The residual contents of SFR had been measured using UV-Vis spectrophotometer at a detection wavelength of 521 nm. All the preformed adsorption tests were replicated three times and the average values were used in the further calculations and investigations with standard deviation less than 4.7% (Ahmed et al., 2024a).

$$q_{e\;\left(mg/g\right)}=\frac{\left(C_{o}-C_{e}\right)V}{m}$$ (1)

The kinetic, as well as the classic isotherm models (Supplementary Table S1), were followed based on the obtained non-linear fitting parameters of both correlation coefficient (R^2^) (Equation 2) and Chi-squared (χ^2^) (Equation 3) (Ahmed et al., 2024a).

$$R^{2}=1-\frac{\sum {\left(q_{e,\exp}-q_{e,cal}\right)}^{2}}{\sum {\left(q_{e,\exp}-q_{e,mean}\right)}^{2}}$$ (2)

$$\chi^{2}=\sum \frac{{\left(q_{e,\exp}-q_{e,cal}\right)}^{2}}{q_{e,cal}}$$ (3)

The nonlinear fitting degrees with the studied advanced isotherm models (Supplementary Table S1) were considered considering the correlation coefficient (R^2^), as well as root mean square error, (RMSE) (Equation 4) (Ahmed et al., 2024a).

$$RMSE=\sqrt{\frac{\sum_{i=1}^{m}{\left({Qi}_{cal}-{Qi}_{\exp}\right)}^{2}}{m^{\prime }-p}}$$ (4)

The m′, p, Qi _cal_, and Qi _exp_ symbols denote the inserted experimental data, number of studied variables, adsorbed quantities of specific pollutant, and experimental adsorbed quantities of the specific pollutant, respectively.

## 3 Results and discussion

### 3.1 Characterization of the adsorbent

**FIGURE 2:**
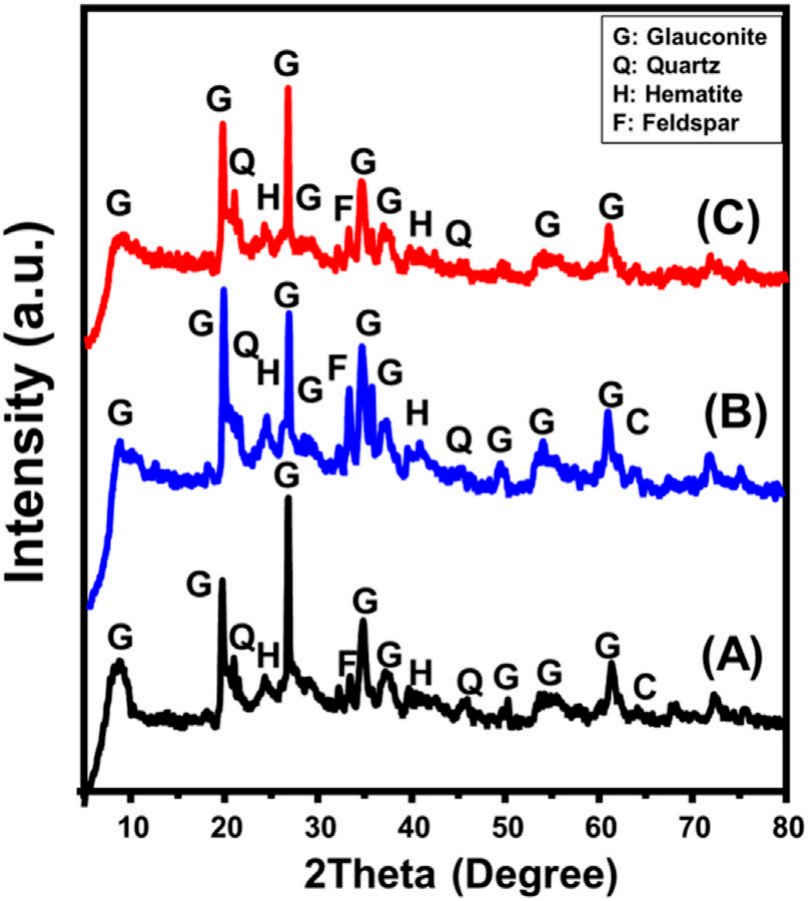
XRD patterns of raw glauconite **(A)**, DMSO modified glauconite **(B)** and exfoliated glauconite **(C)**.

The XRD patterns of both the unprocessed glauconite and synthesized types have been utilized to evaluate the structural alterations and identify the major crystalline phases. The presence of galuconite as the main component, together with contaminants such as quartz, feldspar, calcite, and hematite, has been demonstrated by the observed pattern of unprocessed glauconite (Figure 2A). The glauconite variety that has been realized corresponds to the 1 M-glauconite poly-type exhibiting a well-ordered crystallized framework of the ISII structured class. It can be distinguished by an elevated K_2_O level and exhibits modest swelling characteristics (Baldermann et al., 2022). The presence of glauconite was successfully confirmed by the detectable XRD peaks specified around 8.67^o^, 19.72^o^, 26.70^o^, 34.78^o^, 37.17^o^, and 61.31^o^ possessing a basal spacing value equal to 10.18 Ǻ (Figure 2A) (ICSD 166961) (Baldermann et al., 2022; Abukhadra et al., 2024). For DMSO-treated glauconite, its pattern reveals substantial shifts in the key peaks of glauconite to be located around 8.01^o^, 19.54^o^, 26.50^o^, 34.50^o^, 36.80^o^, and 61.10^o^ (Figure 2B). This confirms the predicted insertion of DMSO molecules as organic reagents between the silicate sheets, alongside the notable deformation that occurs inside the galuconite unit structures. This was further supported by the reported rise in interlayer spacing, which was elevated to 11.2°C, indicating the expansion impact caused by the incorporated DMSO. Similar results were noticed while analyzing the XRD pattern of methoxy glauconite. The primary peaks exhibited significant deviations towards lower situations, specifically at 7.47^o^, 19.52^o^, 26.5^o^, 34.4^o^, and 35.57^o^ (Figure 2C). Additionally, there was a significant expansion impact recognized between the structural silicate units of glauconite, resulting in an increase in basal spacing reaching 11.81 Å.

**FIGURE 3:**
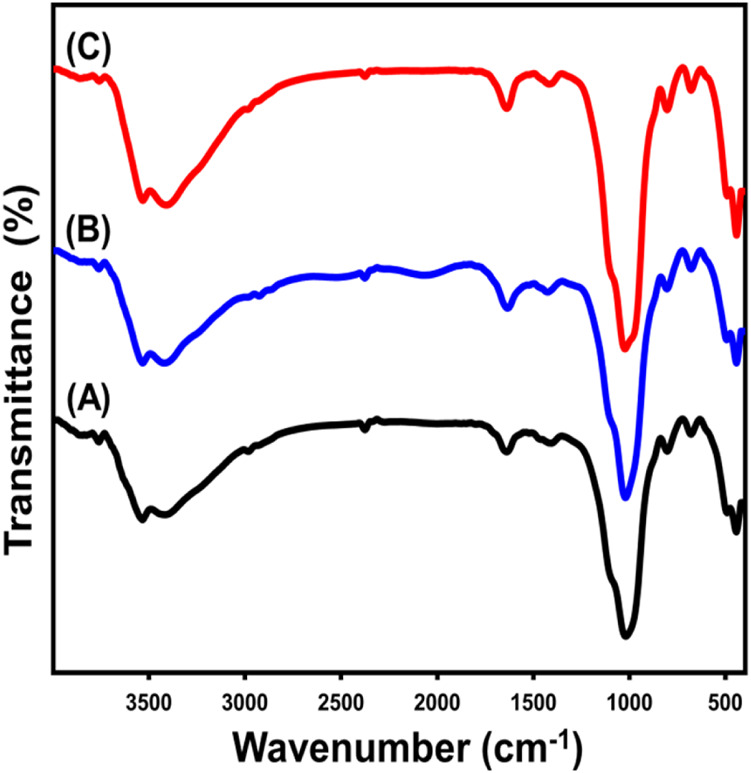
FT-IR spectra of raw glauconite **(A)**, DMSO modified glauconite **(B)** and exfoliated glauconite **(C)** (Allah et al., 2023).

The alterations in the fundamental chemical functionalities throughout the stage of fabrication have been monitored by analyzing the FT-IR spectra of both the original glauconite and the newly produced compounds (Figure 3; Table 1). The spectrum that was determined by analyzing unprocessed glauconite demonstrates its composition as a clay mineral with an aluminosilicate framework (Figure 3A; Table 1). These comprised the verification of the appropriate bands that belong to the Si-O-Si, Si-O, Si(Al)-O-Si, Fe-OH, Si-O-Fe, and OH groups. The hydroxyl functional groups realized at approximately 3,500 cm^-1^ have been correlated with either water being adsorbed or metallic hydroxide compounds embedded in the glauconite framework (Sobeih et al., 2020) (Figure 3A; Table 1). Nevertheless, the hydroxyl groups that have been described to be correlated with the absorption band of approximately 1,600 cm^-1^ represent the unbound water molecules between the layered silicate units, especially in the mixed smectite layers (Moretto et al., 2011; Younes et al., 2021) (Figure 3A; Table 1). Furthermore, the observed bands at 800 cm^-1^ and 490 cm^-1^ reflect the presence of iron metallic elements inside the glauconite composition, which aligns with the results of its XRF analysis. The examined spectra of DMSO-treated glauconite layers and Mth/EXG exhibit identical bands as unprocessed glauconite, without any discernible bands associated with the inserted organic constituents of these reagents (Figures 3B,C; Table 1). The fundamental essential groups’ corresponding bands exhibit a little variation in their specific positions, potentially confirming the structural impact of embedded compounds such as DMSO and alcohol on the crystalline structure of glauconite. Furthermore, there is a little division observed in an identifiable band of Si(Al)-O-Si approximately 1,000 cm^-1^. This confirms the deformation of the glauconite structural units (octahedron along with tetrahedron subunits) due to the partial expansion and separation, or exfoliation, of these layered silicate units (Abdo et al., 2022).

**TABLE 1:** The FT-IR detectable bands of glauconite during the various lateration procedures and the corresponding chemical groups.

Absorption bands (cm^-1^)			Functional chemical groups
Glauconite	DMSO/glauconite	Metheoxy glauconite	
3,532	3,420	3,530	stretching and bending vibrations of –OH groups (Younes et al., 2021)
1,639	1,635	1,639	Interlayer water molecules (Moretto et al., 2011)
1,020	1,022	1,024	Si(Al)–O–Si asymmetric stretching (Sobeih et al., 2020; Allah et al., 2023)
804	806	805	Fe_2_^3+^ OH/Fe^2+^Fe^3+^OH bending (Sobeih et al., 2020)
681	680	681	bending vibration of Si–O and/or -OH (Sobeih et al., 2020)
493	495.6	492	Si–O–Fe^3+^ (Younes et al., 2021; El-Habaak et al., 2016)
445.6	445.02	444	Si–O–Si (Younes et al., 2021; Bruneel et al., 2021)

**FIGURE 4:**
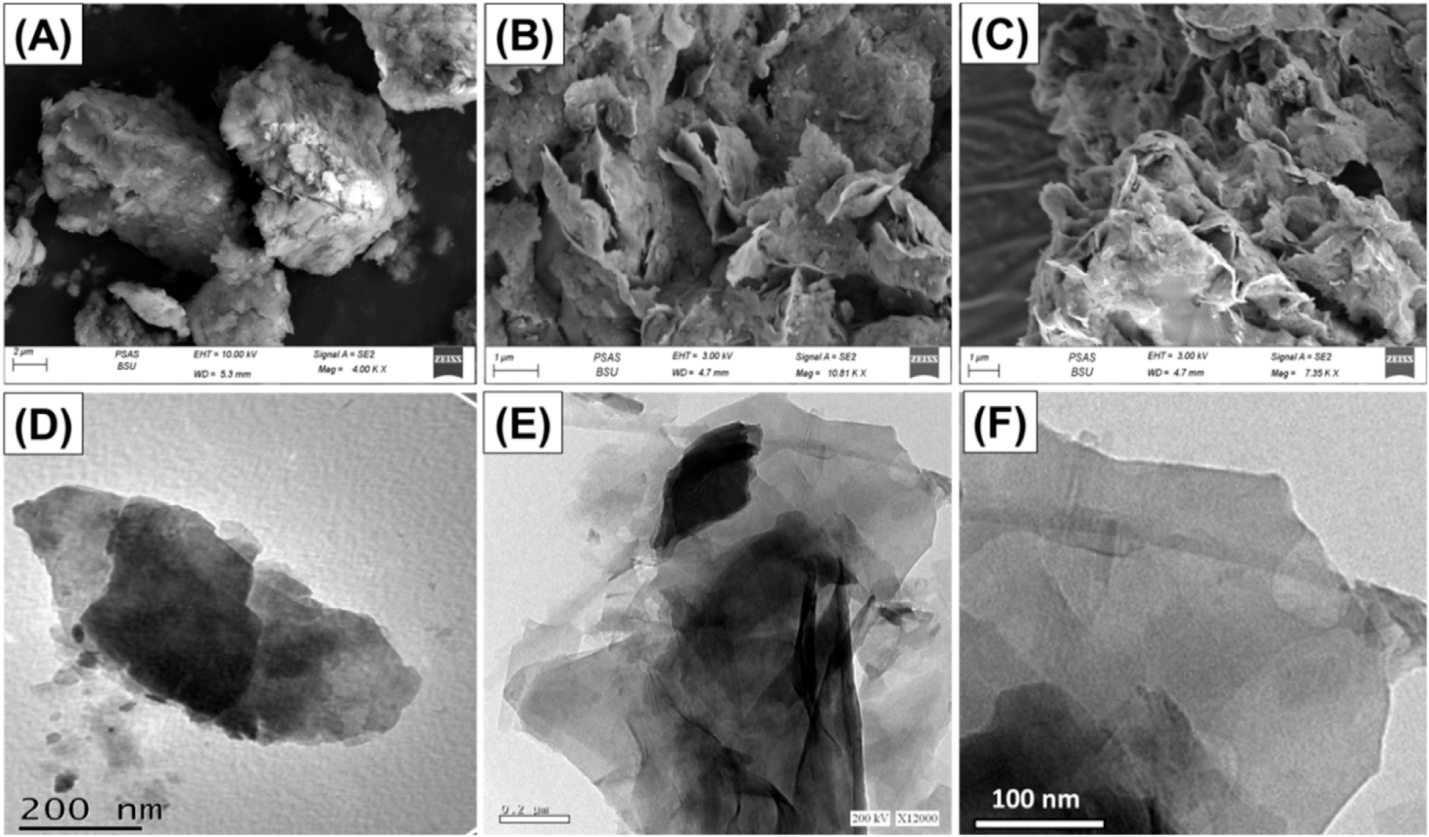
SEM raw glauconite **(A)**, SEM images of exfoliated glauconite **(B and C)**, HRTEM of raw glauconite **(D)**, and HRTEM images of exfoliated glauconite **(E and F)**.

The modifications in the structural characteristics throughout the fabrication procedures of Mth/EXG were monitored using the scanning electron microscopy (SEM) and the high-resolution transmission electron microscopy (HRTEM) photos (Figure 4). The starting unprocessed glauconite has the typical dense and aggregated shape of glauconite, characterized by the frequently observed stacked and compressed sheets (Figure 4A). The insertion of DMSO followed with methanol molecules in between the successive silicate-layered units resulted in considerable peeling and successful separation of them from other layers (Figure 4B). Following the excessive alcohol molecules’ integration, the exfolation effect dramatically intensified, and the glauconite particulates turned into individual layers overlapping one another and comonly forming blended platelets of cornflakes like structures (Figure 4C). The HRTEM photographs of the evaluated materials validate the observed morphologies that were illustrated by the SEM photographs. The glauconite platelets have been observed as massive, almost elliptical granules with no significant interior characteristics (Figure 4D). Following exfoliation, the particles became distinct sheets, with the existence of the blened particulates in cyclinederical form (Figures 4E,F).

### 3.2 Adsorption results

#### 3.2.1 Effect of pH

**FIGURE 5:**
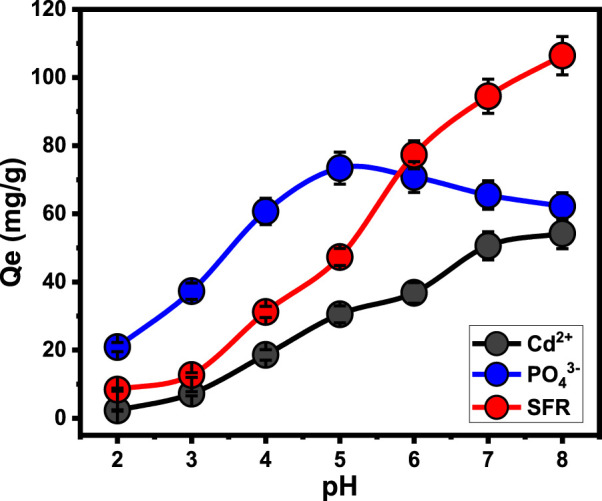
The impact of the solutions, pH on the uptake of PO_4_^3-^, SFR, and Cd^2+^ by Mth/EXG.

The predominant exterior charges and speciation characteristics of the soluble metallic ions are highly dependent upon the pH of the examined aqueous solution (Jiang et al., 2020). The pH impact was followed throughout a range of pH levels that varied from 2 to 8. All other parameters were kept constant at specific levels: an interaction period of 120 min, a temperature of approximately 35°C, a tested volume of 100 mL, a pollutant concentration equal to 100 mg/L, and Mth/EXG dosage of 0.2 g/L. The adsorption results for both SFR and Cd^2+^ demonstrate a significant improvement whenever higher pH levels are tested, reaching pH 8. The adsorption capacities for Cd^2+^ and SFR are 54.2 mg/g and 106.4 mg/g, respectively (Figure 5). The speciation profile of soluble cadmium ions indicates that there are either Cd^2+^ cations or [Cd (H_2_O_6_)]^2+^ cations at a pH around 8 (Jiang et al., 2020). Furthermore, the SFR molecules have been identified as cationic molecules throughout the pH limit that was examined. Moreover, the expected deprotonation of the reactive chemical groups across the exterior of Mth/EXG with the increased basic situations results in the complete saturation of its exterior with hydroxyl ions bearing negative charges. Consequently, the alkaline pH levels provide an ideal environment for powerful electrostatic attractive forces to occur between the water-soluble pollutants (SFR and Cd^2+^) and the exterior of Mth/EXG (Salam et al., 2020; Fu et al., 2018; Li et al., 2023).

The binding of phosphate ions demonstrates varying responses depending on the adapted pH. The highest removal values occurred at pH 5, with a capacity of 73.4 mg/g. However, after pH 5, the effectiveness of Mth/EXG decreased until reaching pH 8 (Figure 5). The ionizing characteristics of soluble phosphate have been determined under the influence of various pH constraints. Within acidic environments (pH < 3), phosphate could be identified as a neutral type (H_3_PO_4_). Within the pH range between 3 and 6, it existed in its acidic H_2_PO^4-^ class. Within neutral and alkaline situations at a pH beyond 6, phosphate existed in two additional acidic types, specifically HPO_4_^2-^ and PO_4_^3-^ (Saleh et al., 2024; Fu et al., 2018). Based on the ionizing behaviors and protonation/deprotonation characteristics of Mth/EXG within the assessed pH levels, it possessed a low uptake effectiveness in acidic environments. This can be explained owing to the protonated exterior of the blended structure, which exhibits weak electrostatic binding to the neutral type of phosphate (H_3_PO_4_). The electrostatic attraction forces steadily increased as the pH increased, leading to the predominance of the acidic phosphate species (H_2_PO_4_^−^) throughout the solution. This resulted in a significant increase in the established capacity of Mth/EXG until pH 5. Further, the deprotonation of the Mth/EXG beneath neutral pH levels led to the formation of negatively charged chemical groups. These groups exhibit significant repellent characteristics with primarily existing phosphate types such as HPO_4_^2-^ and PO_4_^3-^ (Shi et al., 2019; Abukhadra and Mostafa, 2019). The previous finding were supported with the determined values of pH value of zero point charge (pH_(pzc)_). The determined pH_(pzc)_ values during the uptake of PO_4_^3-^, SFR, and Cd^2+^ are pH 6.3, pH 7.2, and pH 6.6, respectively. The surficial charges beyond these values are mainly negative which induce the electrostatic attractions of SFR and Cd^2+^. Also, during pH levels below these values, the surficial charges on the surface of the adsorbent are mainly positive which induce the uptake of PO_4_^3-^ (Neolaka et al., 2018; Neolaka et al., 2021; Neolaka et al., 2022).

#### 3.2.2 Contact time

**FIGURE 6:**
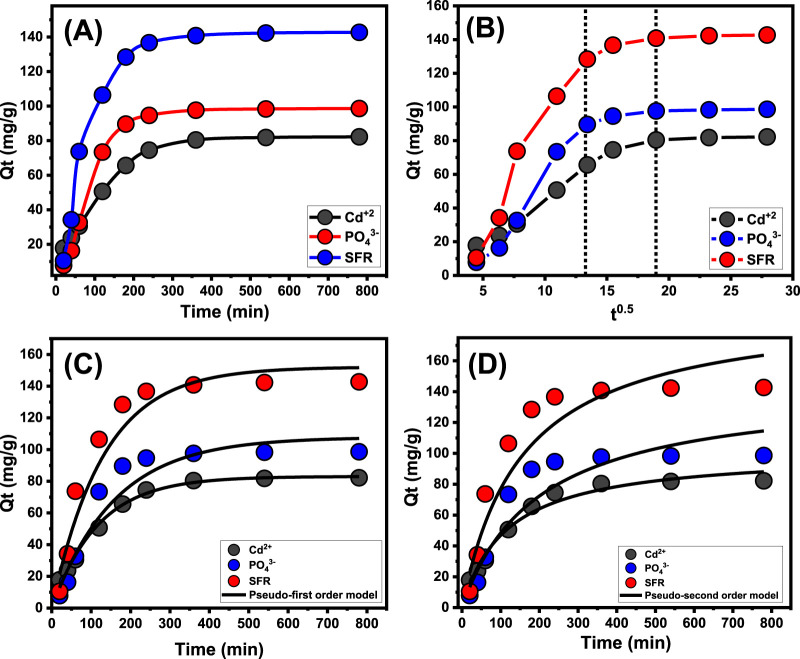
Effect of contact time on the uptake of PO_4_^3-^, SFR, and Cd^2+^ by Mth/EXG **(A)**, Intra-Particle diffusion curves of the uptake processes **(B)**, fitting of the results with Pseudo-First order model **(C)**, and fitting of the results with Pseudo-second order model **(D)**.

An experiment was conducted to examine the retention characteristics of Mth/EXG about the period of the removal of PO_4_^3-^, SFR, and Cd^2+^. The examination’s duration varied from 20 to 780 min. The specific impact of different time intervals was evaluated after the verification of critical parameters, involving the level of pollutants (100 mg/L), temperature (35 °C), volume (100 mL), pH (5 for PO_4_^3-^ and 8 for Cd^2+^ and SFR), and dosage (0.2 g/L). The effectiveness of Mth/EXG in removing PO_4_^3-^, SFR, and Cd^2+^ has been demonstrated by the significant increase in both the quantities of ions adsorbed and the quantifiable rates of removal observed across the studies. Moreover, it is crucial to acknowledge that the time frame of the trials significantly influences the noticed enhancements in the presented uptake features, totaling around 360 min (Figure 6A). Nevertheless, no substantial alterations or improvements have been observed with respect to the rate of elimination of PO_4_^3-^, SFR, and Cd^2+^ ions, nor in the amount of these ions retained beyond the designated contact durations. Previous findings indicate that Mth/EXG may serve as adsorbed agents for PO_4_^3-^, SFR, and Cd^2+^, achieving stability after certain durations. The retention capacities of PO_4_^3-^, SFR, and Cd^2+^ during equilibration using Mth/EXG are 98.6 mg/g, 142.7 mg/g, and 82.2 mg/g, respectively (Figure 6A). The first phases of the assessment indicated substantial improvements and elevated rates of Mth/EXG removal, accompanied by increased amounts of trapped ions. The improvements were ascribed to the readily available interacting and unbound sites throughout the Mth/EXG (Yuan et al., 2024). As the duration of the examination extends, there has been a notable decrease in the total number of accessible sites. The key cause of this behavior is attributed to the prolonged adsorption of PO_4_^3-^, SFR, and Cd^2+^, which fills the previously identified receptors and reduces the overall quantity of available receptors. Consequently, there was a notable decline in the binding rates of PO_4_^3-^, SFR, and Cd^2+^ after a certain duration. Furthermore, the implementation of Mth/EXG demonstrated little enhancement or steady characteristics in the retention of PO_4_^3-^, SFR, and Cd^2+^, indicating equilibrium conditions. The determination of the equilibrium phases of Mth/EXG may be attained by completely saturation of all effective receptors, hence preventing further binding of PO_4_^3-^, SFR, and Cd^2+^ to their interfaces (Salam et al., 2020).

#### 3.2.3 Kinetic studies

##### 3.2.3.1 Intra-particle diffusion behavior

The examination of the intra-particle diffusion pattern may serve as evidence for the mechanical stages and uptake tendencies of PO_4_^3-^, SFR, and Cd^2+^ employing Mth/EXG. The presented curves show three distinct segments with differing slopes (Figure 6B). The analyzed curves exhibit displacements from their initial positions, indicating the simultaneous occurrence of multiple adsorption reactions in conjunction with the diffusion route for PO_4_^3-^, SFR, and Cd^2+^ (Salam et al., 2020; El Qada, 2020). The processes typically consist of three main phases: (1) the interaction between dissolved ions and the unbound binding spots on the surfaces of Mth/EXG (boundary); (2) the layered uptake of dissolve ions (internal retention) and their diffusion characteristics; and (3) the effects of equilibrium and saturation states. (Lin et al., 2021). The first results of this study indicate that the primary mechanisms regulating the binding of PO_4_^3-^, SFR, and Cd^2+^ to the exterior interfaces of Mth/EXG (external retention) were the most significant paths detected at all stages of uptake activities (Figure 6B). The effectiveness of PO_4_^3-^, SFR, and Cd^2+^ uptake during this stage relies on the total number of sites situated across the interfaces of Mth/EXG (Wu et al., 2023). The efficacy of extra-layered adsorption techniques was promptly confirmed by extending the duration until all exterior sites were completely occupied (Figure 6B) (Neolaka et al., 2018; Lin et al., 2021). Additionally, the effects of PO_4_^3-^, SFR, and Cd^2+^ diffusion mechanisms are included throughout this phase. The final processes of PO_4_^3-^, SFR, and Cd^2+^ uptake by Mth/EXG have a considerable influence after equilibrium levels are attained. This indicates that the PO_4_^3-^, SFR, and Cd^2+^, which were efficiently maintained, occupied all available sites (Salam et al., 2020; Sayed et al., 2022). At this step, the elimination of PO_4_^3-^, SFR, and Cd^2+^ is promoted by molecular and interionic attraction processes (Jiang et al., 2020).

##### 3.2.3.2 Kinetic modeling

Modeling the kinetics of binding is crucial for examining time-dependent impacts and comprehending the physical mechanisms involved, including mass transfer and chemical paths that influence adsorption efficiency (Neolaka et al., 2023b). The kinetics of removal pathways for PO_4_^3-^, SFR, and Cd^2+^ were analyzed employing the established kinetic theories based on pseudo-first-order (PFO) and pseudo-second-order (PSO) mathematical models. The PFO modeling was used to elucidate the link between the rates at which the PO_4_^3-^, SFR, and Cd^2+^ ions completely occupy the interaction binding sites, alongside their entire quantities, in order to examine the kinetics of retaining behaviors throughout the equilibrium situations. The PSO concept may demonstrate the correlation between the characteristics of evaluated adsorbents over certain duration. The correlation degrees between the retention characteristics of PO_4_^3-^, SFR, and Cd^2+^ and kinetic concepts were assessed employing nonlinear fitting factors that correspond to the pertinent equations, in relation to both distinctive hypotheses. The appropriate levels of agreement have been determined by the analysis of correlation coefficients (R^2^) and Chi-squared (X^2^) readings (Table 2; Figures 6C, D). The R^2^ and X^2^ values elucidate the basic kinetic characteristics, while the key concepts of the PFO concept provides a better explanation for the bonding processes as well as retention tendencies of PO_4_^3-^, SFR, and Cd^2+^ employing Mth/EXG, in contrast to the evaluated PSO theory. The findings derived from mathematical modeling of the PFO theory indicated that the hypothetical amounts of PO_4_^3-^, SFR, and Cd^2+^ retained were 107.78 mg/g, 152 mg/g, and 83 mg/g, respectively. These values aligned with the actually obtained measurements. The established consistency corroborates the previously acquired findings, which emphasize the superior suitability of the P.F. theory regarding kinetic evaluations (Table 2). According to PFO theory, the primary factors contributing to the adsorbing of PO_4_^3-^, SFR, and Cd^2+^ via Mth/EXG include physical processes, including van der Waals forces and electrostatic attraction (Sherlala et al., 2019; Huang et al., 2018). The analyzed uptake factors further reveal remarkable coherence with the PSO principles; nevertheless, a greater degree of agreement is achieved utilizing the PFO model. Previous studies have demonstrated some chemical reactions, including hydrogen bonding, complexation, and hydrophobic interactions, are likely to enhance or have little effect on the uptake of PO_4_^3-^, SFR, and Cd^2+^ using Mth/EXG (Salam et al., 2020; Sherlala et al., 2019). Physical approaches may generate successive retention layers above the previously established chemically bound PO_4_^3-^, SFR, and Cd^2+^ layers (Jasper et al., 2020).

**TABLE 2:** The mathematical parameters of the evaluated kinetic models.

Kinetic models				
Models	Parameters	PO_4_^3-^	SFR	Cd^2+^
Pseudo-First-order	K_1_ (min^-1^)	0.0063	0.0076	0.0086
	Qe _(Cal)_ (mg/g)	107.78	152.05	83.02
	Qe _(EXP)_ (mg/g)	98.6	142.7	82.2
	R^2^	0.94	0.95	0.98
	X^2^	2.24	3.1	0.25
Pseudo-Second-order	k_2_ (g mg^-1^ min^-1^)	3.55 × 10^−5^	3.31 × 10^−5^	8.53 × 10^−5^
	Qe _(Cal)_ (mg/g)	143.36	195.92	101.15
	Qe _(EXP)_ (mg/g)	98.6	142.7	82.2
	R^2^	0.91	0.91	0.97
	X^2^	3.35	4.8	0.37

#### 3.2.4 Starting concentration

**FIGURE 7:**
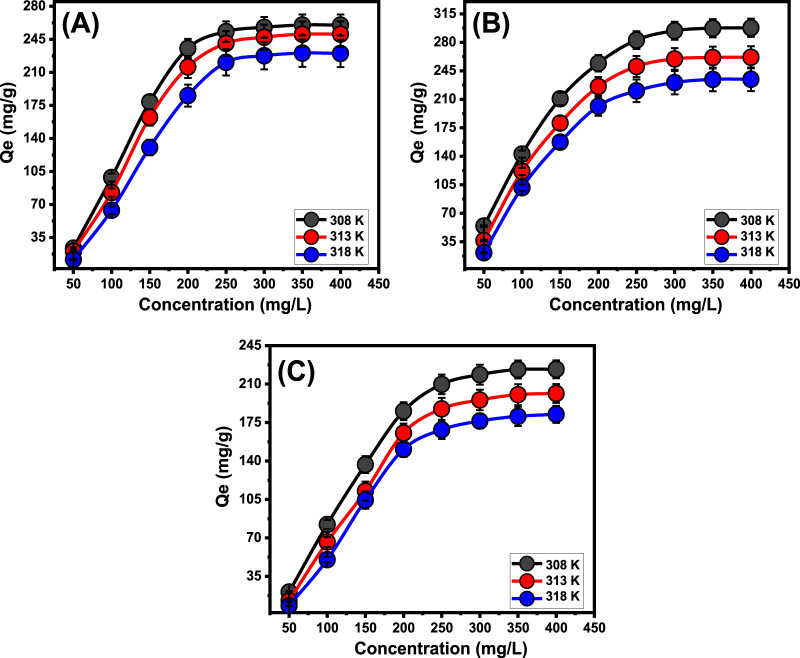
Effect of the starting concentrations on the uptake of PO_4_^3-^ **(A)**, SFR **(B)**, and Cd^2+^ **(C)** by Mth/EXG.

The investigation investigated the impact of initial concentrations of PO_4_^3-^, SFR, and Cd^2+^ to ascertain the maximum elimination activities employing Mth/EXG, alongside related equilibrium conditions, all over the evaluated range of 50–400 mg/L. The supplementary parameters affecting the elimination of PO_4_^3-^, SFR, and Cd^2+^ were maintained at certain levels: an overall volume of 100 mL, a period of 24 h, a dose of 0.2 g/L, and temperatures ranging from 308 K to 318 K. An relationship exists between elevated levels of PO_4_^3-^, SFR, and Cd^2+^ ions and the corresponding rise in their immobilized amounts using Mth/EXG (Figures 7A–C). An elevation in the levels of PO_4_^3-^, SFR, and Cd^2+^ ions inside a certain volume significantly enhanced the diffusion, driving forces, and mobility features of soluble chemical ions. This enhanced the ability to interact with greater amounts of the active retaining sites abundantly present across the exterior of Mth/EXG. The retention effectiveness of PO_4_^3-^, SFR, and Cd^2+^ using Mth/EXG was significantly improved about the evaluated levels of PO_4_^3-^, SFR, and Cd^2+^ (Ahmed et al., 2024b). The relationship is observable only within specified ranges of PO_4_^3-^, SFR, and Cd^2+^ concentrations. Moreover, augmenting the starting levels of PO_4_^3-^, SFR, and Cd^2+^ seems to have little impact on their interaction with Mth/EXG (Figures 7A–C). Determining the equilibrium phases facilitates the determination of optimal retention effectiveness for PO_4_^3-^, SFR, and Cd^2+^. The adsorption abilities of PO43- were quantified as 260 mg/g at 308 K, 250.5 mg/g at 313 K, and 230 mg/g at 318 K (Figure 7A). The observed values for SFR were 297.8 mg/g at 308 K, 261.6 mg/g at 313 K, and 234.6 mg/g at 318 K (Figure 7B). The Cd^2+^ retention levels at temperatures of 308 K, 313 K, and 318 K were 223.4 mg/g, 201.4 mg/g, and 182.3 mg/g, respectively (Figure 7C). The decrease in the uptake of PO_4_^3-^, SFR, and Cd^2+^ observed while using Mth/EXG at different temperatures suggests that the processes under investigation exhibit exothermic characteristics.

#### 3.2.5 Classic isotherm models

**FIGURE 8:**
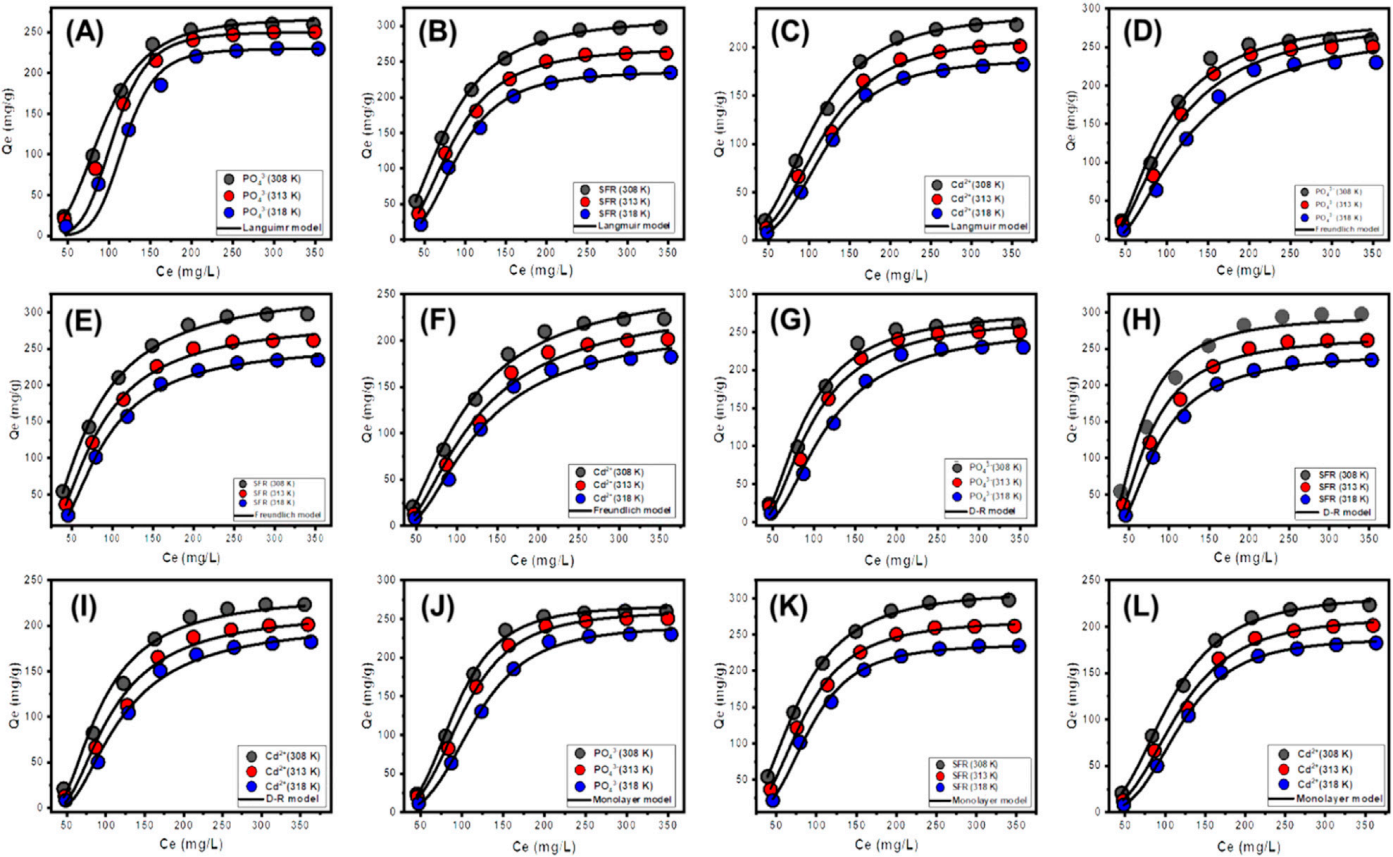
Fitting of the PO_4_^3-^, SFR, and Cd^2+^ uptake results by Mth/EXG with traditional Langmuir model **(A–C)**, traditional Freundlich model **(D–F)**, traditional Dubinin-Radushkevich model **(G–I)**, and advanced monolayer model **(J–L)**.

Traditional equilibrium assessments of adsorption responses were conducted to evaluate the spatial distribution of water-soluble pollutants in aqueous solutions and the materials that adsorb them after stability levels. Conventional equilibrium modeling approaches significantly impact the elucidation of processes. The prevalent isotherm functions provide critical insights into three dimensions: (a) the sorbate’s selectivity for the adsorbent’s reactive surface; (b) the possible amount of soluble chemical ions that may engage with these surfaces; and (c) the ultimate binding features. The binding properties of PO_4_^3-^, SFR, and Cd^2+^ were analyzed for their equilibrium features using the Langmuir (Figures 8A–C), Freundlich (Figures 8D–F), and Dubinin-Radushkevich (D-R) (Figures 8G–I) equilibrium models. The degree of concordance between the proposed equilibrium assumptions outlined in the aforementioned models and the measurable retaining behaviors of PO_4_^3-^, SFR, and Cd^2+^ has been evaluated by non-linear fitting methods. The inquiry included analyzing the correlation coefficient (R^2^) and the Chi-squared (χ^2^) data. Assessment of R^2^ and X^2^ indicates that the Mth/EXG particles have a stronger affinity for binding PO_4_^3-^, SFR, and Cd^2+^, conforming more closely to Langmuir’s concepts than to the Freundlich concept. The equilibrium behavior mentioned above demonstrates that PO_4_^3-^, SFR, and Cd^2+^ display consistent and uniform binding affinities across both the reactive and available sites of Mth/EXG particles. This leads to the formation of monolayers of adsorbed PO_4_^3-^, SFR, and Cd^2+^ ions (Huang et al., 2018; Dawodu et al., 2012). Furthermore, the analysis established that Mth/EXG particulates have favorable adsorption characteristics for PO_4_^3-^, SFR, and Cd^2+^, as shown by the RL values, which are less than 1 (Wu et al., 2023; El Qada, 2020). The mathematical study determined the maximum adsorption capacities (Q_max_) of PO_4_^3-^ as 268.07 mg/g at 308 K, 257.5 mg/g at 313 K, and 234.1 mg/g at 318 K. The predicted values for SFR are 312.3 mg/g at 308 K, 270 mg/g at 313 K, and 237 mg/g at 318 K (Table 3). The calculated concentrations of Cd^2+^ were 234.9 mg/g at 308 K, 211 mg/g at 313 K, and 187.9 mg/g at 318 K (Table 3).

**TABLE 3:** The estimated mathematical parameters of the studied classic equilibrium models.

	Models		308 K	313 K	318 K
Cd^2+^	Langmuir	Q_max_ (mg/g)	234.9	211.1	187.9
		Q_EXP_ (mg/g)	223.4	201.4	182.3
		b(L/mg)	2.02 × 10^−5^	5.14 × 10^−5^	5.71 × 10^−6^
		R^2^	0.998	0.997	0.999
		X^2^	0.106	0.274	0.649
	Freundlich model	1/n	0.62	0.58	0.54
		k_F_ (mg/g)	265.8	243.5	219.5
		R^2^	0.984	0.983	0.981
		X^2^	0.74	0.61	0.82
	D-R model	β (mol^2^/KJ^2^)	0.0046	0.0054	0.0062
		Q_m_ (mg/g)	233.6	215.6	202.6
		R^2^	0.984	0.986	0.989
		X^2^	1.357	1.258	0.998
		E (KJ/mol)	10.42	9.62	8.98
PO_4_^3-^	Langmuir model	Q_max_ (mg/g)	268.07	257.5	234.1
		Q_EXP_ (mg/g)	260	150.5	230
		b(L/mg)	2.36 × 10^−5^	2.71 × 10^−5^	1.42 × 10^−6^
		R^2^	0.998	0.955	0.994
		X^2^	0.160	4.77	5.96
	Freundlich model	1/n	0.49	0.54	0.54
		k_F_ (mg/g)	291.4	293.4	280.77
		R^2^	0.990	0.987	0.989
		X^2^	1.013	1.366	1.22
	D-R model	β (mol^2^/KJ^2^)	0.0044	0.0049	0.0059
		Q_m_ (mg/g)	280.95	272.40	258.92
		R^2^	0.99	0.98	0.99
		X^2^	1.039	1.662	1.420
		E (KJ/mol)	10.66	10.10	9.20
SFR	Langmuir model	Q_max_ (mg/g)	312.3	270.13	237.14
		Q_EXP_ (mg/g)	297.8	261.6	234.6
		b(L/mg)	4.73 × 10^−5^	6.52 × 10^−5^	5.54 × 10^−6^
		R^2^	0.999	0.998	0.997
		X^2^	0.041	0.118	0.121
	Freundlich model	1/n	0.62	0.55	0.49
		k_F_ (mg/g)	336.42	289.84	255.08
		R^2^	0.97	0.987	0.978
		X^2^	0.189	0.277	0.386
	D-R model	β (mol^2^/KJ^2^)	0.012	0.017	0.022
		Q_m_ (mg/g)	296.9	268.2	246.1
		R^2^	0.97	0.99	0.99
		X^2^	1.51	0.69	0.218
		E (KJ/mol)	6.45	5.43	4.76

The equilibrium parameters of the D-R hypothesis provide a comprehensive understanding of the energy variations shown by Mth/EXG nanoparticles throughout the purification processes of PO_4_^3-^, SFR, and Cd^2+^, irrespective of the particle’s degree of heterogeneity or homogeneity depending on Gaussian energy distribution (El Qada, 2020). The examination of the D-R modeling results provides critical insights into the calculation of free adsorption energy (E) and its relevance in understanding fundamental mechanisms, regardless of whether they are chemical or physical in nature. The energy levels related to retaining activities may be categorized into three separate groups: below 8 kJ/mol, between 8 and 16 kJ/mol, and beyond 16 kJ/mol. At these energy levels, the primary mechanisms consist mostly of significant physical interactions, weak chemical reactions, or a combination of physical and chemical processes, together with significant chemical activities (Sayed et al., 2022). The determined E values for SFR uptake procedures using Mth/EXG were below the permissible energy limits (below 8 kJ/mol) for the physical processes (Table 3). The established levels for PO_4_^3-^ and Cd^2+^ align with the hypothesized range for weak chemical or complicated interactions between chemical and physical reactions (8–16 kJ/mol) (Table 3).

#### 3.2.6 Advanced isotherm models

Employing statistical physics methods to mimic the equilibrium characteristics of adsorption activities can give an in-depth investigation of the distinctive characteristics of the processes. This study uses mathematical models to assess the interactions between water-soluble pollutants and external reactive chemical groups present as binding spots onto adsorbent interfaces. The mathematical formulas used in this investigation provide calculated parameters that accurately describe the principal activities, such as energetic and steric aspects. The simulations include several steric factors, notably Nm, which denotes the total number of occupied adsorption sites over the contact surface of Mth/EXG. The estimations also include the quantity of held ions (n) within an individual receptor and the maximum retention efficacy of PO_4_^3-^, SFR, and Cd^2+^ using Mth/EXG at full saturation (Q_sat_). The evaluated energetic parameters, derived from the model’s fitting variables, include internal energy (E_int_), entropy (Sa), retention energies (E), and free enthalpy (G). Non-linear regression analyses have been used to examine the aforementioned hypotheses of the established models. The prior inquiry was effectively conducted using multivariable nonlinear regression algorithms with the Levenberg-Marquardt iterative approach. The derived fitting values were later used to evaluate and describe the adsorption processes of PO_4_^3-^, SFR, and Cd^2+^ by Mth/EXG. The analysis was completed using a closely analogous model, namely the monolayer model of a single active site (Figures 8J–L). Table 4 displays the calculated variables and the fitting quality.

**TABLE 4:** The steric and energetic parameters of the evaluated advanced isotherm models.

Material	Parameters	Values		
		308K	313K	318K
Cd^2+^	n	2.81	3.04	3.48
	Nm (mg/g)	83.45	69.26	53.89
	Q_sat_ (mg/g)	234.5	210.5	187.5
	C_1/2_ (mg/L)	105.2	115.4	119.3
	ΔE (kJ/mol)	−12.56	−12.87	−13.12
PO_4_^3-^	n	3.27	3.37	3.44
	Nm (mg/g)	82.54	77.35	70.02
	Q_sat_ (mg/g)	269.9	260.7	240.8
	C_1/2_ (mg/L)	92.55	102.1	116.5
	ΔE (kJ/mol)	−7.59	−7.83	−8.10
SFR	n	2.29	2.69	3.19
	Nm (mg/g)	136.3	100.2	74.3
	Q_sat_ (mg/g)	312.1	269.6	237
	C_1/2_ (mg/L)	77.34	83.96	91.25
	ΔE (kJ/mol)	−4.83	−5.0	−5.18

##### 3.2.6.1 Steric properties

###### 3.2.6.1.1 Number of adsorbed Se ions per site (n)

**FIGURE 9:**
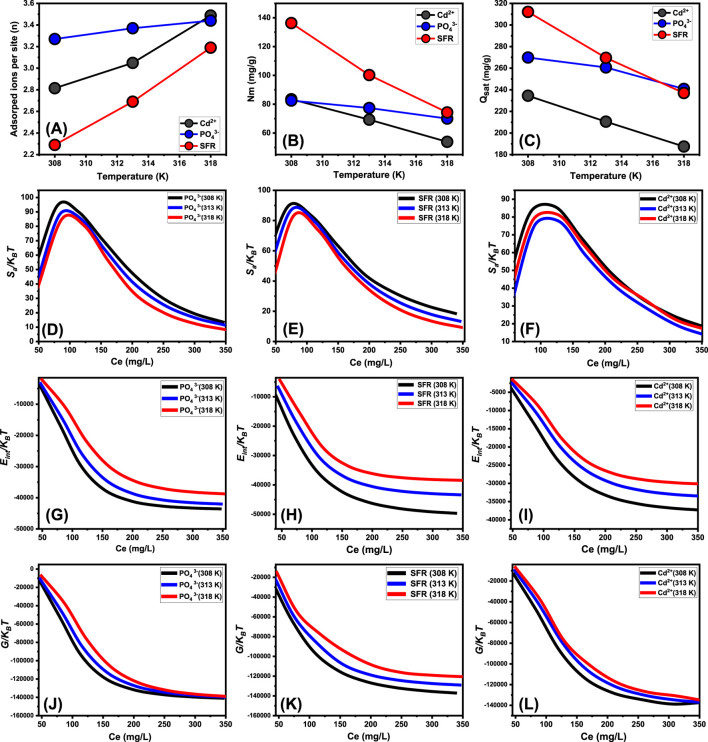
The changes in the steric (number of adsorbed ions per site **(A)**, occupies sites density **(B)**, and saturation uptake capacity **(C)**) and thermodynamic functions [entropy **(D–F)**, internal energy **(G–I)**, and enthalpy **(J–L)**] during the uptake of PO_4_^3-^, SFR, and Cd^2+^ by Mth/EXG.

The mathematical outcomes of the n (Se) function provide strong proof on the configurational properties of the immobilized PO_4_^3-^, SFR, and Cd^2+^ ions across the exterior interfaces of Mth/EXG. This includes either vertical or horizontal arrangements. Moreover, these findings are significant for understanding the processes that regulate binding reactions, including many dockings or interactions. The binding of PO_4_^3-^, SFR, and Cd^2+^ ions via various retention sites is significantly affected by multi-anchorage or multi-docking interactions. Adsorption mechanisms displaying values below 1 relate to the horizontal positioning of these ions. Conversely, characteristics above a level of 1 indicate the existence of PO_4_^3-^, SFR, and Cd^2+^ in non-parallel and vertical orientation. The removal mechanisms of such systems are primarily facilitated by multi-ionic processes, whereby a single site may accommodate several ions (Sayed et al., 2022; Mobarak et al., 2021). The calculated values of n, indicating the total number of ions trapped by a single uptake site onto the exterior of Mth/EXG, range from 3.27 to 3.44 for PO_4_^3-^, 2.29 to 3.19 for SFR, and from 2.8 to 3.48 for Cd^2+^ (Figure 9A). The total number of PO_4_^3-^, SFR, and Cd^2+^ ions present at each site are higher than 1. Consequently, PO_4_^3-^, SFR, and Cd^2+^ were sequestered by a multi-ionic interactions mechanism. Every retention site inside the Mth/EXG particulates had the ability to accommodate multiple ions, organized in vertical configurations with non-parallel characteristics. The binding sites across the exterior surface of Mth/EXG might each hold a maximum of four ions of PO43-, SFR, and Cd^2+^. The estimated n levels of Mth/EXG for PO_4_^3-^, SFR, and Cd^2+^ show a significant increase with increasing operation temperature from 308 K to 318 K (Figure 9A). This realization emphasizes that the aggregation affinities of PO_4_^3-^, SFR, and Cd^2+^ ions tend to increase across the surface of the composite at elevated temperature. Furthermore, this implies that thermal activating steps occur prior to the binding of these ions by the Mth/EXG particles (Hsu et al., 2024; Dhaouadi et al., 2021).

###### 3.2.6.1.2 Density of the active sites (Nm)

The overall number of PO_4_^3-^, SFR, and Cd^2+^ filled receptors (Nm) in the outermost layer of Mth/EXG particulates throughout all stages may be accurately assessed by analyzing the density of active sites for PO_4_^3-^, SFR, and Cd^2+^ (Figure 8B). The measured values for the computed Nm of Mth/EXG at different temperatures for PO_4_^3-^ are 82.54 mg/g at 308 K, 77.35 mg/g at 313 K, and 70.02 mg/g at 318 K (Figure 9B). The calculated levels for SFR were 136.3 mg/g at 308 K, 100.2 mg/g at 313 K, and 84.3 mg/g at 318 K. Furthermore, the recorded levels of Cd^2+^ were 83.45 mg/g at 308 K, 69.26 mg/g at 313 K, and 53.89 mg/g at 318 K (Figure 9B). The determined Nm levels associated with the SFR binding processes are greater than what has been reported for Cd^2+^ and PO_4_^3-^, thus explaining its highest adsorption characteristics in the previously presented tests. This finding further supports the existence of additional chemical groups across the exterior of Mth/EXG particulates, which possess a remarkable chemical reactivity to form complexes with the chemical framework of SFR dye.

Temperature-sensitive reversible variations in the Nm levels of PO_4_^3-^, SFR, and Cd^2+^ have been observed in relation to the impact of temperature (Figure 9B). The findings align with the earlier recognized trends in n, as the enhanced aggregation properties of PO_4_^3-^, SFR, and Cd^2+^ lead to a reduction in the total number of filled sites. This could be elucidated by the influence of temperature on the activity levels of existing retention sites engaged in the activities (Hsu et al., 2024; Sellaoui et al., 2020). The study highlights the detrimental effects of temperature increase on the number of functional sites, either via the potential deactivation of specific sites or the reduction in the duration necessary for certain sites to effectively absorb and retain PO_4_^3-^, SFR, and Cd^2+^. Prior experiments indicated similar patterns, maybe resulting from the expected dispersion of adsorbed ions or their desorption from the materials to which they were bonded. The desorption behavior resulted from the reduction in saturation restrictions of warm solutions (Ahmed et al., 2024a).

###### 3.2.6.1.3 Adsorption capacity at the saturation state of (Q_sat_)

The fully saturated binding features of Mth/EXG (Qsat) offer excellent retention of PO_4_^3-^, SFR, and Cd^2+^, together with enhanced tolerance. The estimate of Qsat amounts is determined by two primary factors: the designated density of occupied sites (Nm) and the cumulative number of PO_4_^3-^, SFR, and Cd^2+^ ions kept by each individual site (n). The Mth/EXG’s ability to hold onto PO_4_^3-^ is 269.9 mg/g at 308 K, 260.7 mg/g at 313 K, and 240.8 mg/g at 318 K (Figure 9C). The highest possible adsorption qualities of SFR were calculated as 312.1 mg/g at 308 K, 269.6 mg/g at 313 K, and 237 mg/g at 318 K (Figure 9C). The Mth/EXG hybrid had the highest Cd^2+^ adsorption capacity, with values of 234.5 mg/g at 303 K, 210.5 mg/g at 313 K, and 187.5 mg/g at 318 K (Figure 9C). The adverse impacts of temperature signify the exothermic nature of the PO_4_^3-^, SFR, and Cd^2+^ retention activities using Mth/EXG. The results demonstrate that greater uptake temperatures result in increased thermal collisions, which subsequently diminish the binding efficacy of PO_4_^3-^, SFR, and Cd^2+^ (Mobarak et al., 2021). Moreover, the temperature-dependent observable traits of Q_sat_ show similarities to the behavior characterized by Nm rather than n (Figure 8C). The findings indicate that the number of interacting receptors, rather than the unique binding affinity of each individual receptor, is the primary determinant influencing the retention efficacy of PO_4_^3-^, SFR, and Cd^2+^ by Mth/EXG.

##### 3.2.6.2 Energetic properties

###### 3.2.6.2.1 Adsorption energy

The quantified energy fluctuations (ΔE) across the adsorption processes of PO_4_^3-^, SFR, and Cd^2+^ may provide valuable insights into fundamental processes, regardless of their association with physical or chemical processes. Physical reactions show energies below 40 kJ/mol, while chemical pathways reveal energetic levels over 80 kJ/mol. The binding energies serve as significant criteria for classifying different mechanistic responses in physical processes. Various physical processes may be classified based on the magnitude of binding energy. These binding interactions include hydrogen bonds (less than 30 kJ/mol), dipole bonds (2–29 kJ/mol), van der Waals forces (4–10 kJ/mol), electrostatic binding (2–50 kJ/mol), and hydrophobic interacting (5 kJ/mol). The calculation of removal energies (ΔE) for PO_4_^3-^, SFR, and Cd^2+^ was conducted using Equation 5. This equation incorporates the solubility of PO_4_^3-^, SFR, and Cd^2+^ (S), the gas constant (R = 0.008314 kJ/mol·K), the levels of these ions under half-saturation conditions of Mth/EXG, and a designated temperature (T) (Dhaouadi et al., 2020).

$$∆E=RT\;\ln\left(\frac{S}{C}\right)$$ (5)

**FIGURE 10:**
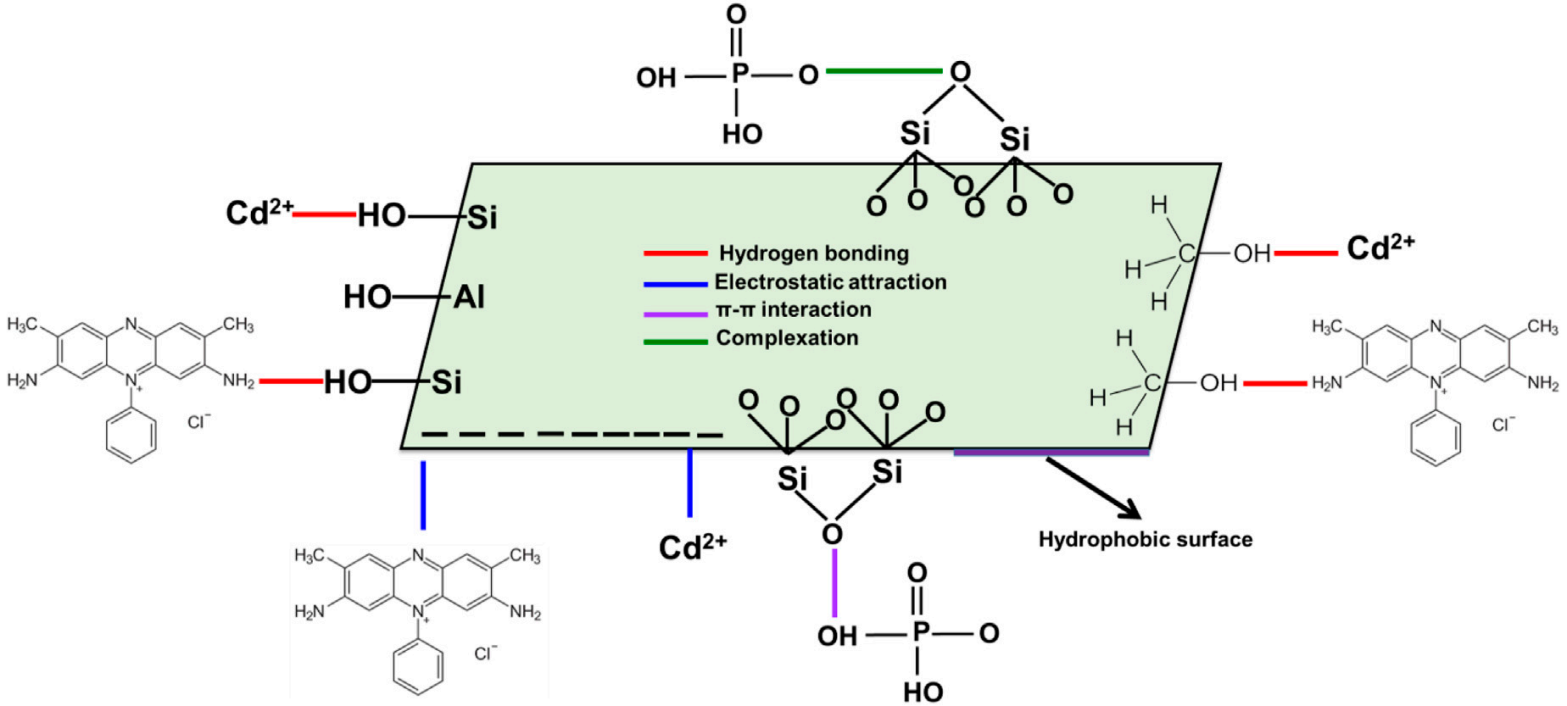
Schematic diagram for the adsorption mechanisms of Cd^2+^, PO_4_^3-,^ and SFR by Mth/EXG.

The adsorption energy levels of PO_4_^3-^, SFR, and Cd^2+^ by Mth/EXG vary from −7.59 kJ/mol to −8.10 kJ/mol, −4.83 kJ/mol to −5.18 kJ/mol, and −12.56 kJ/mol to −13.12 kJ/mol, respectively (Table 4). These values are within the determined boundaries for physisorption processes. The measured values for PO_4_^3-^ and SFR indicate that hydrogen bonds along with electrostatic attraction, dipole forces and van der Waals forces are the key processes involved in their removal by Mth/EXG. The binding process of Cd^2+^ via Mth/EXG is regulated mainly by electrostatic attraction, van der Waals forces, and hydrogen bonds as demonstrated from the computed results. The suggested uptake mechanisms by Mth/EXG were represented schematically in Figure 10. The negative signs of the ∆E results reveal that the Cd^2+^, PO_4_^3-^, and SFR adsorption processes by Mth/EXG are characterized by their exothermic features.

##### 3.2.6.3 Thermodynamic functions

###### 3.2.6.3.1 Entropy

The entropy (Sa) associated with retaining procedures of PO_4_^3-^, SFR, and Cd^2+^ utilizing Mth/EXG clearly illustrates the order and disorder behaviors that characterize their exterior interfaces under varied levels of PO_4_^3-^, SFR, and Cd^2+^ ions, as well as differing temperature conditions. The properties of Sa have been illustrated by using the results derived from Equation 6, which included the already determined values for Nm and n, together with the anticipated concentrations during the half-saturation phases of Mth/EXG (C1/2) (Sellaoui et al., 2016).

$$\frac{S_{a}}{K_{B}}=Nm\left\{\ln\left(1+{\left(\frac{C}{C_{\frac{1}{2}}}\right)}^{n}\right)-n{\left(\frac{C}{C_{\frac{1}{2}}}\right)}^{n}\;\frac{\ln\left(\frac{C}{C_{\frac{1}{2}}}\right)}{1+{\left(\frac{C}{C_{\frac{1}{2}}}\right)}^{n\;}}\;\right\}$$ (6)

The analysis of the resultant graphs reveals a notable decrease in entropy levels (Sa) upon the adsorption of PO_4_^3-^, SFR, and Cd^2+^ using Mth/EXG, especially at higher concentrations (Figures 9D–F). Observations reveal a discernible decline in the disorder features of Mth/EXG surfaces when the levels of PO_4_^3-^, SFR, and Cd^2+^ increase. The entropy characteristics further enhance the effective docking and binding of PO_4_^3-^, SFR, and Cd^2+^ to vacant and significant binding sites situated on the Mth/EXG surface, even at extremely low starting levels (Sellaoui et al., 2020; Dhaouadi et al., 2020). The highest entropy values at equilibrium have been established at concentrations of 80.23 mg/L (308 K), 83.48 mg/L (313 K), and 87.24 mg/L (318 K) throughout the adsorption of PO_4_^3-^ (Figure 9D). The maximum level of entropy was matched by the equilibrium contents for SFR, which were 71.46 mg/L at 308 K, 75.7 mg/L at 313 K, and 79.7 mg/L at 318 K (Figure 9E). The concentrations associated with maximal entropy throughout Cd^2+^ adsorption are 122.6 mg/L at 308 K, 127.5 mg/L at 313 K, and 129 mg/L at 318 K (Figure 9F). The levels measured following half-saturation phases of Mth/EXG closely resemble these equilibrium values. Thus, the existence of leftover binding sites hinders the docking of additional ions. Furthermore, the significant reductions observed in the evaluated entropy levels indicate a substantial decline in the number of available sites, together with a marked drop in the mobility and diffusion properties of the PO_4_^3-^, SFR, and Cd^2+^ ions (Sellaoui et al., 2016).

###### 3.2.6.3.2 Internal energy and free enthalpy

The experiment examined the internal energy (E_int_) associated with the chemical interactions of PO_4_^3-^, SFR, and Cd^2+^ with Mth/EXG. The investigation examined the properties of free enthalpy (G) and the effects of varying levels of PO_4_^3-^, SFR, and Cd^2+^, as well as operating temperature, on these properties. The assessment was performed using Equations 7 and 8, which calculated the results based on the predetermined values for Nm, n, and C1/2, together with the translational partition (Zv) (Dhaouadi et al., 2021).

$$\frac{E_{\mathrm{int}}}{K_{B}T\;}=n\;N_{m}\left[\left(\;\frac{{\left(\frac{C}{C_{1/2}}\right)}^{n}\;\ln\left(\frac{C}{Z_{v}}\right)}{1+{\left(\frac{C}{C_{1/2}}\right)}^{n}}\right)-\left(\;\frac{n\ln\left(\frac{C}{C_{1/2}}\right)\;{\left(\frac{C}{C_{1/2}}\right)}^{n}}{1+{\left(\frac{C}{C_{1/2}}\right)}^{n}}\right)\right]$$ (7)

$$\frac{G}{K_{B}T\;}=n\;N_{m}\frac{\ln\left(\frac{C}{Z_{v}}\right)}{1+{\left(\frac{C_{1/2}}{C}\right)}^{n}}$$ (8)

The analysis of Eint fluctuations related to the removal activities of PO_4_^3-^, SFR, and Cd^2+^, using Mth/EXG, reveals negative values. The results indicate a significant decrease in Eint when the temperature increases between 308 K and 318 K (Figures 9G–I). This examination confirms the spontaneous and exothermic properties of the Mth/EXG retaining procedures using Mth/EXG. The enthalpy readings and activities exhibit similar characteristics, which correspond to those of the internal energy. The G data exhibit negative trends and demonstrate a reversible relationship with the tested temperature (Figures 9I–K). This signifies a decrease in feasibility aspects and corroborates the exothermic nature and spontaneous characteristics of the retaining of PO_4_^3-^, SFR, and Cd^2+^ ions employing Mth/EXG.

#### 3.2.7 Recyclability

**FIGURE 11:**
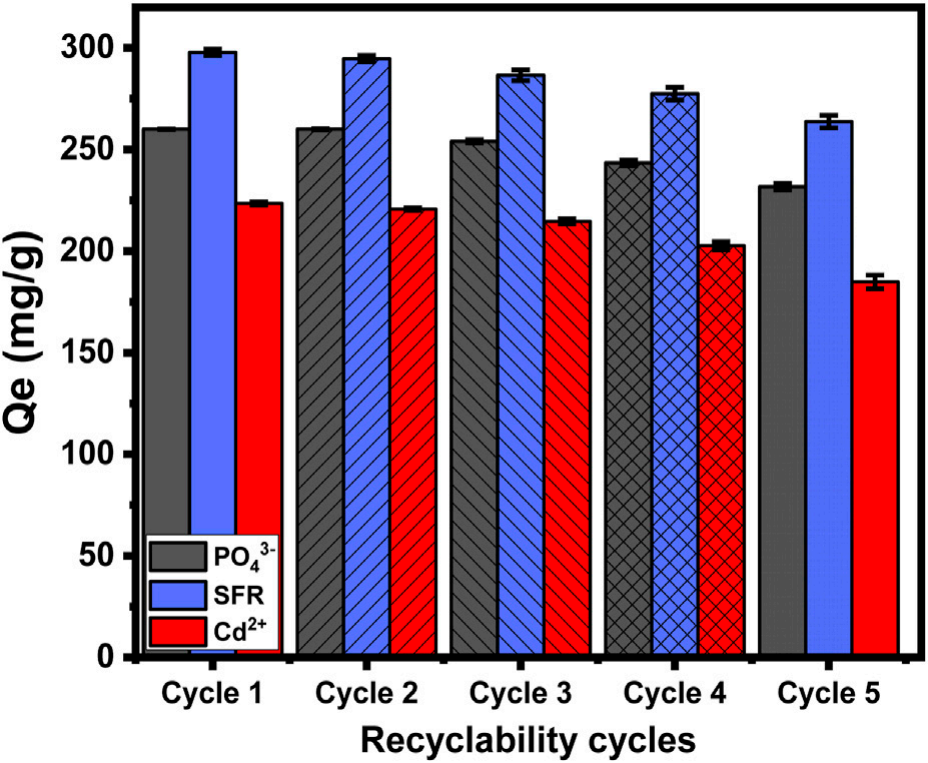
Recyclability properties of Mth/EXG during the adsorption of PO_4_^3-^, SFR, and Cd^2+^.

The recycling and reuse potential of Mth/EXG as an adsorption material is a significant factor when evaluating its qualities for practical and large-scale water treatment activities. The residual particulates of Mth/EXG, obtained after conducting adsorption experiments for PO_4_^3-^, SFR, and Cd^2+^, were subjected to a 10-min washing process utilizing distilled water. This washing process has been repeated four times. Subsequently, the cleansed Mth/EXG particulates had been carefully dried inside an electric oven at a temperature of 55 °C for duration of 8 h and then used in subsequent adsorption cycles. The recyclable adsorption experiments of PO_4_^3-^, SFR, and Cd^2+^ have been performed, implementing specific values of pH (pH 5 for PO_4_^3-^ and pH 8 for Cd^2+^ and SFR), Mth/EXG dose (0.2 g/L), investigated levels (400 mg/L), interaction time (24 h), temperature (308 K), and volume (100 mL) (Figure 11). The results of the five recycling experiments demonstrate the excellent stability and potential to be reused of Mth/EXG particulates for successful use in removal operations throughout the five cycles (Figure 11). The amounts of PO_4_^3-^ adsorbed throughout the examined rounds are as follows: 260 mg/g (Cycle 1), 260 mg/g (Cycle 2), 254 mg/g (Cycle 3), 243.4 mg/g (Cycle 4), and 231.7 mg/g (Cycle 5). The binding levels of SFR are as follows: 297.8 mg/g (Cycle 1), 294.7 mg/g (Cycle 2), 286.5 mg/g (Cycle 3), 277.5 mg/g (Cycle 4), and 263.7 mg/g (Cycle 5) (Figure 11). The adsorption amounts of Cd^2+^ during the examined rounds are as follows: 223.4 mg/g (Cycle 1), 220.6 mg/g (Cycle 2), 214.6 mg/g (Cycle 3), 202.6 mg/g (Cycle 4), and 184.8 mg/g (Cycle 5) (Figure 11). The noticeable reduction in the effectiveness of Mth/EXG after repeat reusing runs may be attributed to the ongoing development of a complex among the ions that were absorbed, together with the chemical composition of the composite material.

Glauconite has a highly environmental clay structure and is commonly used as fertilizer for its content of potassium and iron. The synthetic glauconite-based adsorbents display remarkable recyclability to be used several times, and after the complete exhaustion of the structure, each spent product after the adsorption of each pollutant can be disposed of and recycled by different techniques. The spent sample after the adsorption of phosphate can be applied directly as fertilizer. Following cadmium adsorption, the exhausted material can potentially be utilized for recovering the cadmium ions using acidic washing as well as magnetic extraction, and the remaining materials can be repurposed as an adsorbent on its own or in combination with other types of structures. Furthermore, the structure may undergo heat treatment or microwave exposure, resulting in a cadmium oxide-supported EXG or cadmium iron silicate framework suitable for many applications. After safranin adsorption, the spent structure can undergo carbonization or activation, resulting in a type of activated carbon/EXG composite suitable for various applications.

#### 3.2.8 Effect of coexisting ions

**FIGURE 12:**
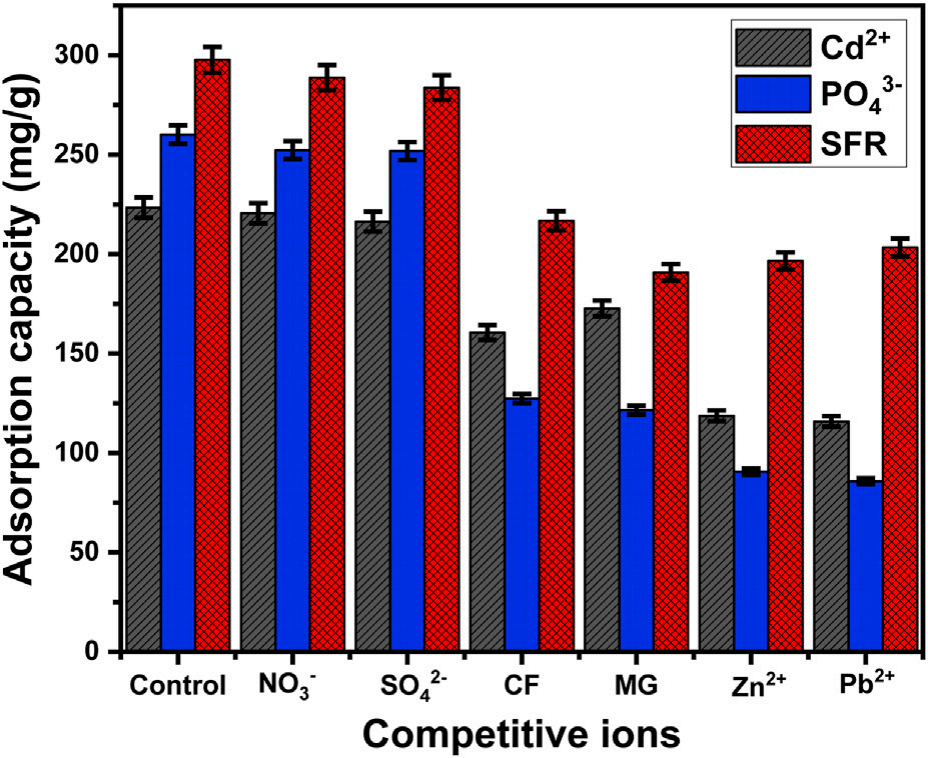
Effect of the coexisting ions on the affinities of Mth/EXG during the adsorption of PO_4_^3-^, SFR, and Cd^2+^.

The impact of several species of soluble chemicals, including Pb^2+^, Zn^2+^, NO_3_^−^, SO_4_^2-^, malachite green dye (MG), and chlorpyrifos insecticide (CF), on the binding selectivity of the Mth/EXG towards Cd^2+^, PO_4_^3-^, and SFR ions has been investigated. The adsorption experiments for Cd^2+^, PO_4_^3-^, and SFR were done, taking into account particular levels of pH (pH 5 for PO_4_^3-^ and pH 8 for Cd^2+^ and SFR), concentrations (400 mg/L), Mth/EXG dose (0.2 g/L), interaction duration (24 h), temperature (308 K), and volume (100 mL) (Figure 12). The presence of the soluble chemical anions (NO_3_^−^ and SO_4_^2-^) does not significantly affect the removal of Cd^2+^, PO_4_^3-^, and SFR ions using Mth/EXG (Figure 12). This assignment has been assigned to the types of chemical complexes formed as well as the exchange of ligands. The uptake of NO_3_^−^ along with SO_4_^2-^ occurred by forming outer-sphere complexes, which have lower stability compared to the inner-sphere complexes described for ions of the studied pollutants (Saleh et al., 2024). In relation to the presence of different types of organic pollutants (MG and CF), they had a significant competitive effect, particularly during the adsorption of Cd^2+^ and PO_4_^3-^. The quantity of Cd^2+^ and PO_4_^3-^ adsorbed decreased to 160.6 mg/g and 127.4 mg/g, respectively, in the presence of CF and to 172.7 mg/g (Cd^2+^) and 121.6 mg/g (PO_4_^3-^) in the presence of MG (Figure 12). In terms of the influence of MG and CF on the uptake of SFR, they have a considerable competitive influence on the binding potential of Mth/EXG for SFR. However, the amount of SFR adsorbed is still greater than the established levels of MG and CF. Furthermore, the existence of metallic cations such as Pb^2+^ and Zn^2+^ had a significant competitive effect on the removal of Cd^2+^, PO_4_^3-^, and SFR using Mth/EXG. This was evident from the decrease in the amount of their adsorbed ions. Nevertheless, the Mth/EXG particulates maintain considerable capacities for Cd^2+^, PO_4_^3-^, and SFR retention, regardless of being interacting with the other metallic ions of Pb^2+^ and Zn^2+^ (Figure 12). The overall results confirm that Mth/EXG is suitable for successfully eliminating Cd^2+^, PO_4_^3-^, and SFR ions, irrespective of whether there are other soluble chemicals, including different anions, cations, and organic compounds. Furthermore, the findings given demonstrate the efficacy of Mth/EXG for removing various types of organic and inorganic contaminants from water.

#### 3.2.9 Comparison study

The determined adsorption capacities of Mth/EXG as adsorbent for PO_4_^3-^, SFR, and Cd^2+^ were compared with studied organic and inorganic adsorbents in literature in addition to the raw galuconite and exfoliated glauconite (Supplementary Table S2). The recognized best capacities of raw galuconite are 91.7 mg/g (PO_4_^3-^), 82.5 mg/g (Cd^2+^), and 104.8 mg/g (SFR). For the exfoliated product prior to the modification with methanol (EXG) the determined values are 196.4 mg/g (PO_4_^3-^), 183.7 mg/g (Cd^2+^), and 260.3 mg/g (SFR) (Supplementary Table S2). Such results demonstrate the impact of the exfoliation followed by methanol modification in inducing the uptake efficacy of the studied pollutants. Also, the presented values reflected the higher adsorption properties of the Mth/EXG composite as compared most of the presented materials in the table (Supplementary Table S2). Therefore, the synthetic structure is a suitable and effective product to be applied as a low-cost and environmental adsorbent in the realistic purification of wastewater from different species of dissolved organic and inorganic chemicals as pollutants. The structure is less expensive than the aforementioned adsorbents, and its uses may attain excellent removal effectiveness with little dosages and in brief timeframes. Furthermore, it is suggested as a non-toxic natural environmental material with extensive applications as a fertilizer.

## 4 Conclusion

Natural glauconite was successfully separated into silicate nano-sheets, functionalized with methanol (Mth/EXG), and applied in enhanced remediation of phosphate (PO_4_^3-^), safranin dye (SFR), and cadmium (Cd^2+^) in an aqueous environment. The product displayed uptake capacities of 269.9 mg/g (PO_4_^3-^), 312 mg/g (SFR), and 234.5 mg/g (Cd^2+^). The uptake mechanisms and behaviors were followed based on the estimated parameters of the advanced isotherm modeling applying a monolayer model of one energy site. However, each site across Mth/EXG can be filled with 4 ions of these pollutants in vertical orientation, the surface display changes in the quantities of the reacting sties during their uptake (Nm = 82.5 mg/g (PO_4_^3-^), 136.3 mg/g (SFR), and 83.4 mg/g (Cd^2+^)). The uptake of these ions involved mainly multi-ionic physical mechanisms of exothermic and spontaneous behaviors, considering the adsorption energy (less than 40 kJ/mol), free adsorption energy of D-R model (8–16 kJ/mol), thermodynamic functions, and steric parameters. The methoxy modified version of EXG showed remarkable selectivity towards the target pollutants regardless the impact of coexisting inorganic and organic soluble chemicals as coexisting ions.

## Data Availability

The original contributions presented in the study are included in the article/Supplementary Material, further inquiries can be directed to the corresponding author.
