# Supplementary material for: Experimental and advanced equilibrium studies on the enhanced adsorption of phosphate, cadmium, and safranin dye pollutants using methoxy exfoliated glauconite
Source: Front Chem. 2024 Nov 6;12:1471994. doi: 10.3389/fchem.2024.1471994 (PMC11576185; doi:10.3389/fchem.2024.1471994)
Supplement: Supplementary file 1 [file Table1.docx]

Table.S1. Nonlinear equations of kinetic, classic isotherm, and advanced isotherm models

| Kinetic models | | |
| --- | --- | --- |
| Model | Equation | Parameters |
| Pseudo-first-order | $Q_{t}=Q_{e} (1-e^{{-k}_{1}.t})$ | Q_t_ (mg/g) is the adsorbed ions at time (t), and K_1_ is the rate constant of the first-order adsorption (1/min) |
| Pseudo-second-order | $Q_{t}=\frac{Q_{e}^{2}k_{2}t}{1+Q_{e}k_{2}t}$ | Qe is the quantity of adsorbed ions after equilibration (mg/g), and K_2_ is the model rate constant (g/mg min). |
| Classic Isotherm models | | |
| Model | Equation | Parameters |
| Langmuir | $Q_{e}=\frac{Q_{max} bC_{e}}{(1+bC_{e})}$ | *C_e_* is the rest ions concentrations (mg/L), *Q_max_* is the theoritical maximum adsorption capacity (mg/g), and *b* is the Langmuir constant (L/mg) |
| Freundlich | $Q_{e}=K_{f}C_{e}^{1/n}$ | K_F_ (mg/g) is the constant of Freundlich model related to the adsorption capacity and n is the constant of Freundlich model related to the adsorption intensities |
| Dubinin–Radushkevich | $Q_{e}=Q_{m}e^{-\betaɛ^{2}}$ | β (mol^2^/KJ^2^) is the D-R constant, ɛ (KJ^2^/mol^2^) is the polanyil potential, and Q_m_ is the adsorption capacity (mg/g) |
| Advanced isotherm models | | |
| Model | Equation | Parameters |
| Monolayer model with one energy site (Model 1) | $Q_{e}=nN_{o} =\frac{nN_{M}}{1+{(\frac{C1/2}{C_{e}})}^{n}}=\frac{Q_{o}}{1+{(\frac{C1/2}{C_{e}})}^{n}}$ | Qe is the adsorbed quantities in mg/g  n is the number of adsorbed ion per site  Nm is the density of the effective receptor sites (mg/g)  Q_o_ is the adsorption capacity at the saturation state in mg/g  *C_e_* is the rest ions concentrations (mg/L)  C1/2 is the concentration of the ions at half saturation stage in mg/L  C1 and C2 are the concentrations of the ions at the half saturation stage for the first active sites and the second active sites, respectively  n1 and n2 are the adsorbed ions per site for the first active sites and the second active sites, respectively |
| Monolayer model with two energy sites (Model 2) | $Q=\frac{n_{1}N_{1M}}{1+{(\frac{C_{1}}{C})}^{n_{1}}}+\frac{n_{2}N_{2M}}{1+{(\frac{C_{2}}{C})}^{n_{2}}}$ |  |
| Double layer model with one energy site (Model 3) | $Q=Q_{o}\frac{({\frac{C}{C1/2})}^{n}+2({\frac{C}{C1/2})}^{2n}}{1+({\frac{C}{C1/2})}^{n}+({\frac{C}{C1/2})}^{2n}}$ |  |
| Double layer model with two energy sites (Model 3) | $Q=Q_{o}\frac{({\frac{C}{C1})}^{n}+2({\frac{C}{C2})}^{2n}}{1+({\frac{C}{C1})}^{n}+({\frac{C}{C2})}^{2n}}$ |  |

**Table.S2.** Comparison study between the developed Mth/EXG as adsorbent and other adsorbents in literature

| Adsorbent | q_max_ (mg/g) | References |
| --- | --- | --- |
| Cd^2+^ | | |
| Maghemite modified MWCNTs | 78.18 | Gatabi et al., (2016) |
| Magnetic functionalized MCM-48 | 114.08 | Anbia et al., (2015) |
| Fe_3_O_4_-chitosan@bentonite | 62.1 | Feng et al., (2019) |
| Attapulgite/CoFe_2_O_4_@SiO_2_-chitosan/EDTA | 127.79 | Wang et al., (2020) |
| Nano-hydroxyapatite /chitosan | 123.5 | Cui et al., (2016) |
| Composite chitosan biosorbent | 108.7 | Madala et al., (2017) |
| Amino functional SBA-15 | 93.3 | Aguado et al., (2009) |
| Cross-linked magnetic Chitosan-phenylthiourea resin | 120 | Monier and Abdel-Latif, (2012) |
| MCM-41 modified with thioglycolic acid | 91.3 | Kenawy et al., (2018) |
| Ti-MCM-48 | 83.57 | Chen et al., (2011) |
| Chitosan/vermiculite | 58.48 | Chen et al., (2018) |
| Raw glauconite | **82.5** | **This study** |
| EXG | **183.7** | **This study** |
| Mth/EXG | **234.5** | **This study** |
| SFR | | |
| Ppy NF/Zn-Fe LDH | 63.4 | Mohamed et al., (2018) |
| Glass-MCM-48 | 62.5 | Abukhadra et al., (2019) |
| MCM-41 | 68.8 | Kaur et al., (2015) |
| MgO-FLG-FE | 201.1 | Reddy et al., (2018) |
| CuO-NP | 189.5 | Vidovix et al., (2021) |
| N/porous graphite | 20.66 | Shaban et al., (2017) |
| Sepiolite | 233.81 | Barhdadi et al., (2024) |
| Ferruginous kaolinite | 59.3 | Debnath et al., (2017) |
| Raw glauconite | **104.8** | **This study** |
| EXG | **260.3** | **This study** |
| Mth/EXG | **312** | **This study** |
| PO_4_^3-^ | | |
| Lanthanum hydroxides | 107.5 | Xie et al., (2014) |
| La doping magnetic graphene | 116.28 | Nodeh et al., (2017) |
| Biochar | 133 | Yao et al., (2011) |
| Mg(OH)_2_/ZrO_2_ | 87.2 | Lin et al., (2019) |
| Zirconia/graphite oxide | 149.3 | Zong et al., (2013) |
| ZrO_2_ nanoparticles | 99 | Su et al., (2013) |
| La_100_SBA-15 | 45.6 | Yang et al., (2011) |
| Hydrous zirconium oxide | 51.8 | Lin et al., (2017) |
| Calcined Mg-Al-LDHs | 40.78 | Das et al., (2006) |
| Kaolintic clay | 38.46 | Hamdi and Srasra, 2012 |
| Raw glauconite | **91.7** | **This study** |
| EXG | **196.4** | **This study** |
| Mth/EXG | **269.9** | **This study** |
